# Environmental immobility: A systematic review of empirical research

**DOI:** 10.1007/s13280-025-02195-9

**Published:** 2025-06-11

**Authors:** Moitrayee Sengupta

**Affiliations:** Chair of Environmental Development and Risk Management, Faculty of Environmental Sciences, TUD Dresden University of Technology, 01062 Dresden, Germany

**Keywords:** Climate change, Environmental change, Environmental migration, Environmental non-migration, Immobility, Trapped populations

## Abstract

Environmental migration research has traditionally focused on *mobility* outcomes in the face of environmental change. However, the ‘trapped populations’ concept introduced in the influential Foresight Report (2011) drew attention to *immobility* outcomes owing to people’s inability or unwillingness to migrate away from environmentally high-risk settings. To assess the evolution of the empirical research landscape of environmental immobility, this paper systematically reviews 55 studies in terms of their thematic focus areas, theoretical and methodological concerns, geographical distribution, the interplay between environmental and societal contexts, the characterisation of immobility processes, and interrelations with mobility processes. The findings suggest a predominant focus on the causes of immobility based on a dichotomisation of immobility as ‘forced’ and ‘voluntary’. Using insights from migration theory, this paper provides a schematic framework that helps us think analytically about environmental immobility processes in terms of a continuum of choices and constraints and implications for stayers’ well-being.

**Supplementary Information:**

The online version contains supplementary material available at 10.1007/s13280-025-02195-9.

## Introduction

Environmental migration (McLeman and Gemenne 2018) or mobilities (Wiegel et al. 2019) research has traditionally concerned itself with the impacts of environmental change on various human *mobility* outcomes, including forced displacements due to extreme weather events (McAdam 2010), ‘voluntary’ forms of migration^1^ for adapting to the impacts of slow-onset environmental change (Warner and Afifi 2014), and the planned relocation of people in the event of reduced habitability of places (Ferris and Weerasinghe 2020). Breaking with this predominant focus on mobility, an influential 2011 study under the UK Government Office for Science (Foresight 2011) identified people unable to migrate, or *immobility* outcomes in the face of environmental change, to be a previously overlooked category warranting scientific and policy attention (Ayeb-Karlsson et al. 2018). Termed ‘trapped populations’ or those involuntarily immobile, researchers identified those lacking the *ability* to migrate away from adverse environmental conditions, despite having the *willingness* and the *need* to do so, to be a particularly vulnerable population group (Black and Collyer 2014; Zickgraf 2018). People were found to be ‘trapped’ in environmentally harmful settings due to a range of constraints on movement, including low financial resources, restrictive migration policies, place-based obligations, and societal gender norms (Ayeb‐Karlsson et al. 2022). Studies also found people *choosing to stay put* under environmental stress due to cultural and relational attachments to place and trust in local knowledge systems (Farbotko and McMichael 2019; Mallick and Schanze 2020).

Environmental immobility research has helped put a previously invisible population group on the academic radar in migration scholarship (Zickgraf 2018). Understanding staying processes is key to supporting people living at environmental risks. While some researchers suggest that those ‘trapped’ in place due to a lack of resources to move to safer locations are more vulnerable to risks (Black and Collyer 2014), voluntary stayers who lack sufficient information on the severity of environmental threats (Zickgraf 2018) or lack viable alternatives to staying^2^ (Straehle 2023) may constitute a similarly vulnerable population group.

Much before research on immobility gained ground in settings impacted by environmental change,^3^ human geographers were already examining immobility processes, albeit in contexts not directly linked with biophysical and climatic factors (Gruber 2021).^4^ Their interest in considering immobility outcomes emerged in view of a ‘mobility bias’ animating the migration studies research field, which prioritised the study of human movement as an exceptional feature of an otherwise apparently sedentary human society, concomitantly neglecting the study of staying intentions (Schewel 2020). Breaking with this research tradition, scholars began examining the obverse, i.e. the causes and consequences of immobility in instances where mobility represents the norm and immobility proves exceptional. They sought to understand why people stay in place despite migration potentially bearing greater socioeconomic advantages or where it constitutes a normalised livelihood strategy, a social tradition, or a cultural rite of passage (e.g. see the edited issue by Stockdale and Haartsen 2018; Debray et al. 2023).

The aspiration-capability framework, initially developed to explain the immobility outcomes of a community in Cape Verde with widespread yet unfulfilled migration aspirations (Carling 2002), provides conceptual orientation to immobility research in human geography. It proposes that people’s unrealised aspirations to migrate may be a function of capability constraints arising from mobility barriers such as restrictive migration policies or the lack of access to economic and social capital, thereby leading to *involuntary immobility*. In contrast, people may *willingly stay put* owing to factors that ‘retain’ them in place, such as location-specific advantages and attachments or that ‘repel’ them from anticipated negative migration experiences and outcomes in destination areas (Schewel 2020). Staying as a conscious and deliberate choice is distinguished from more passive forms of staying, where people’s limited capability to migrate may hinder the development of migration intentions, resulting in *acquiescent immobility* (Schewel 2015; Carling and Schewel 2018).^5^ Immobility researchers also point to how intrahousehold relationships of power influence migration and staying processes, such as the ‘enabling’ immobility of some household members that facilitate the migratory projects of others by the former’s assumption of household responsibilities, or some household members being ‘left behind’ due to entrenched societal norms that encourage the mobility of one group, e.g. able-bodied men, over that of others (Jónsson 2011; Mata-Codesal 2015, 2018).

Environmental immobility^6^ scholarship has similarly followed course by distinguishing between *voluntary* and *involuntary immobility* outcomes, with the former characterising *desired immobility* despite environmental risks, while the latter points to *constraints on desired mobility* in the face of risks. Existing reviews which map the evolution of research on this topic largely focus on involuntary immobility processes. For instance, Ayeb-Karlsson and colleagues trace discursive narratives surrounding the ‘trapped populations’ concept in academic literature (Ayeb-Karlsson et al. 2018) and subsequently explore how the ‘trapped’ figure is characterised in academic and climate policy texts (portrayed either as a ‘human security threat’ or a ‘victim’ requiring saving) and the empirical and institutional geographies of study (Ayeb‐Karlsson et al. 2022).

**Table 1:** Search terms used with Boolean operators. The three research concerns were combined using the Boolean operator ‘AND’

Facets of the research concern	Search terms	Search terms with Boolean operators
Environmental and climate change	Environmental change, climate change, climate variability, global warming, slow-onset event, extreme weather event, flood, drought, storm, cyclone, wildfire, extreme temperature, increasing temperature, desertification, loss of biodiversity, land and forest degradation, glacial retreat and related impacts, ocean acidification, sea-level rise, and salinisation	“environmental change” OR “climat* change” OR “climat* variability” OR “global warming” OR “slow-onset event*” OR “extreme weather event*” OR flood* OR drought OR storm* OR cyclon* OR wildfire OR “extreme temperature*” OR “increasing temperature*” OR desertification OR “biodiversity loss” OR “land and forest degradation” OR “glacial retreat” OR “ocean acidification” OR “sea-level rise” OR salinisation
Immobility	Immobility, non-migration, staying, trapped populations, immobile, non-migrants, stayers, left behind	immobil* OR “non-migra*” OR trapped* OR stay* OR “left behind”
Referent	Humans, people, persons, inhabitants	human* OR people OR person* OR inhabitant*

Other review papers show how theoretical approaches from allied fields can help conceptualise environmental immobility processes. In a special issue edited by Czaika and Reinprecht (2022), Mallick and Hunter (2023) show how approaches in behavioural and cognitive sciences, such as decision heuristics, can explain staying aspirations among people exposed to environmental threats. Zickgraf (2021) indicates how approaches from migration theory, such as the aspiration-capability framework, new economics of labour migration (NELM), and the mobilities paradigm, can be integrated to better explain immobility drivers and relations with mobility practices. Additionally, reviews with a regional focus explore contextual drivers of environmental immobility, such as in the African context (Balgah and Kimengsi 2022).

Scholars also outline conceptual approaches to studying environmental immobility. For instance, Mallick and Schanze (2020) and Pemberton et al. (2021) recommend conceptually grounding the study of voluntary immobility in theoretical ideas of resilience, with the latter emphasising the need to explore issues of power, marginalisation, and translocality in voluntary staying decisions. Employing the mobilities paradigm, Boas et al. (2022) examine the plurality of environmental immobility processes by showing how mobility and immobility practices can intersect in complex ways instead of being fixed and discrete categories. To historically situate immobility processes, researchers outline an intergenerational approach that focuses on how communities transfer mobility and immobility norms across generations and implement historical knowledge systems of coping with recurring risks (Mallick and Hunter 2024; Mallick and van den Berg 2025). Scholars also propose intersectional approaches that account for vulnerabilities faced by people belonging to different social groupings, the implications of life-course transitions, and gender hierarchies that encourage the mobility of some while limiting that of others (Cundill et al. 2021).

Building on these reviews, this paper assesses the empirical research landscape in environmental immobility scholarship. It systematically reviews the thematic areas of empirical scholarship, the theoretical and methodological approaches employed, the geographical distribution of research, the interplay of environmental and societal contexts, the characterisation of immobility processes in the literature, and the interrelations between immobility and mobility practices. Using conceptual insights from migration theory, it goes on to delineate intermedial immobility categories beyond the forced/voluntary divide that account for a range of choices and constraints that may intersect in immobility decision-making under environmental stress.

## Review methodology

Systematic literature reviews provide an analysis of the state of knowledge in a research field or given topic using systematic and explicit methods. This review follows the PRISMA (Preferred Reporting Items for Systematic Reviews and Meta-Analyses) approach, which was first developed in the field of medicine (Moher et al. 2009) and was subsequently adapted for use in environmental and climate change studies (Berrang-Ford et al. 2011) and, more particularly, in the environmental migration research field (Obokata et al. 2014; Ghosh and Orchiston 2022; Mortreux et al. 2023; Nabong et al. 2023). It involves developing a set of review questions, developing a search strategy, developing the selection criteria for studies, selecting studies using the selection criteria, analysing studies, and synthesising results.

**Fig. 1:**
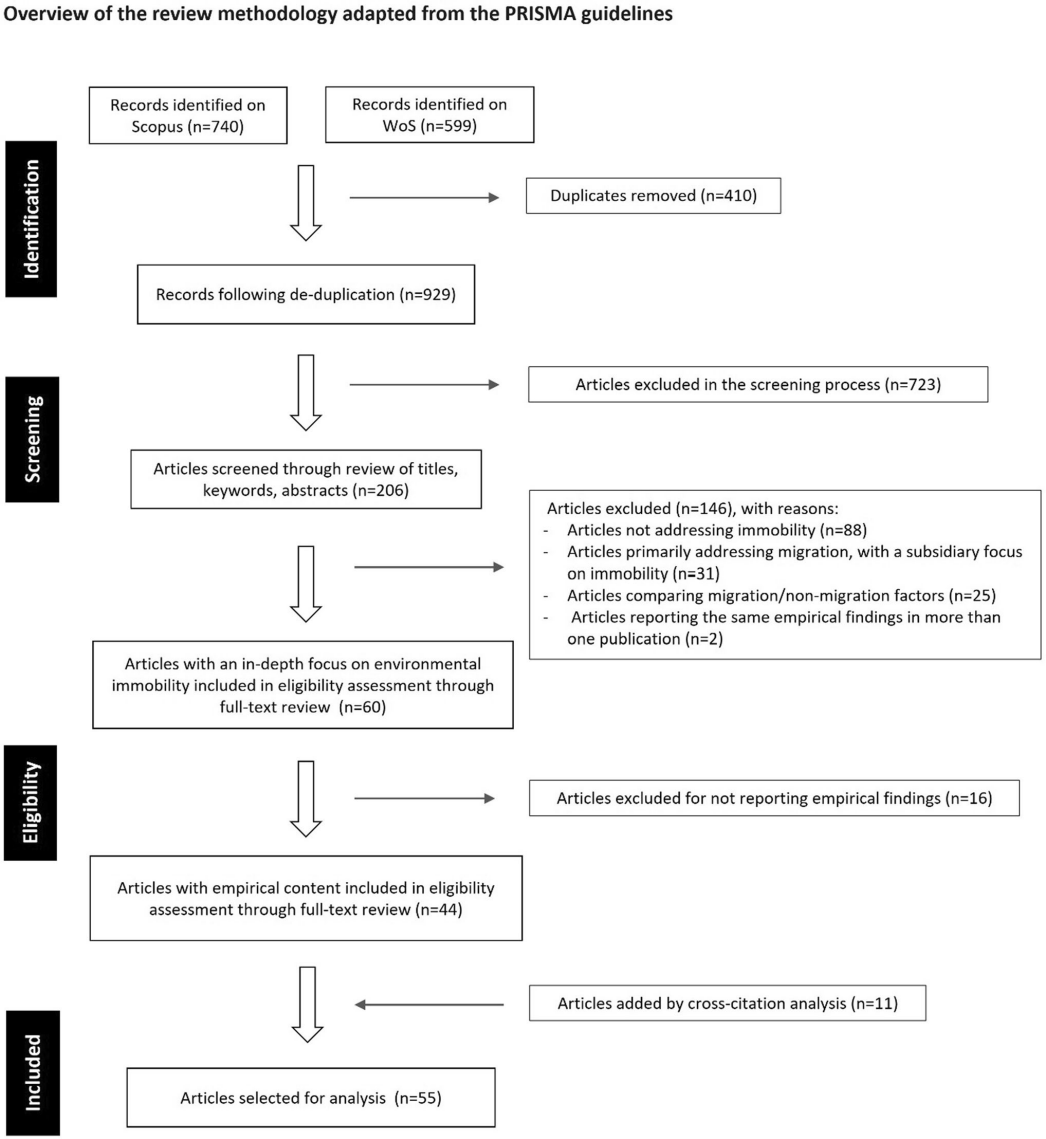
Overview of the systematic literature review methodology adapted from the PRISMA guidelines

A repository of peer-reviewed journal articles^7^ reporting empirical findings on environmental immobility was developed. A literature search was conducted on the Scopus and Web of Science databases in February 2024 to identify articles to be used in the analysis. The article search was limited from the year 2011, which is when the concept of ‘trapped populations’ was first developed in the Foresight Report (2011), following which research on environmental immobility took off.^8^ The search strategy involved using Boolean search terms representing the key concerns of the review paper, i.e. the influence of environmental and climatic change on human immobility processes (Table 1).^9^ The search was limited to English-language journal articles^10^ and excluded unrelated subject areas from the engineering, medical, and natural sciences. Following de-duplication, the search yielded an initial result of 929 articles from the two literature databases (Fig. 1).

The article selection process was undertaken through the two stages of screening and eligibility assessment. In the screening stage, articles were screened by manually reviewing titles, keywords, and abstracts to identify those that deal with the broad field of environmental/climatic mobility and immobility. In the eligibility assessment stage, the screened articles were read to identify those that could be included in the review based on two inclusion criteria. *First, only those articles that exclusively focus on environmental immobility were included*. These articles incorporate the study of environmental immobility ‘directly and intentionally’ (Zickgraf 2018, 74) in their stated research questions and research design, and their key focus is to provide an in-depth understanding of the context-specific immobility processes.^11^*Second, only those articles that report empirical findings were included.* Accordingly, publications where authors collect primary data or apply new analytical lenses to previously collected data were selected.^12^ Following the eligibility assessment stage, relevant articles from the references of the selected papers which met the inclusion and exclusion criteria but were not originally captured in the electronic search results were added to the review.^13^ This led to a final sample of 55 articles to be considered in the review.

The analytic strategy was adapted from the two qualitative data analysis strategies of qualitative content analysis (Schreier 2014) and thematic analysis (Braun and Clarke 2021) to produce a synthesis of the reviewed literature. Qualitative content analysis involves developing a coding frame to analyse data comprising coding categories developed either inductively from the data, deductively from existing concepts or research questions, or by a combination of both. Here, a coding frame was developed deductively from the review questions posed in the introductory section. Once the structure of the coding frame was developed, corresponding notes were maintained for each article to address the review questions using spreadsheet software, resulting in the generation of a ‘text matrix’ (Schreier 2014, 180). This text matrix was thematically analysed to capture the emerging research trajectories in the considered literature.

## Assessing the empirical research landscape

### Thematic focus areas

**Fig. 2:**
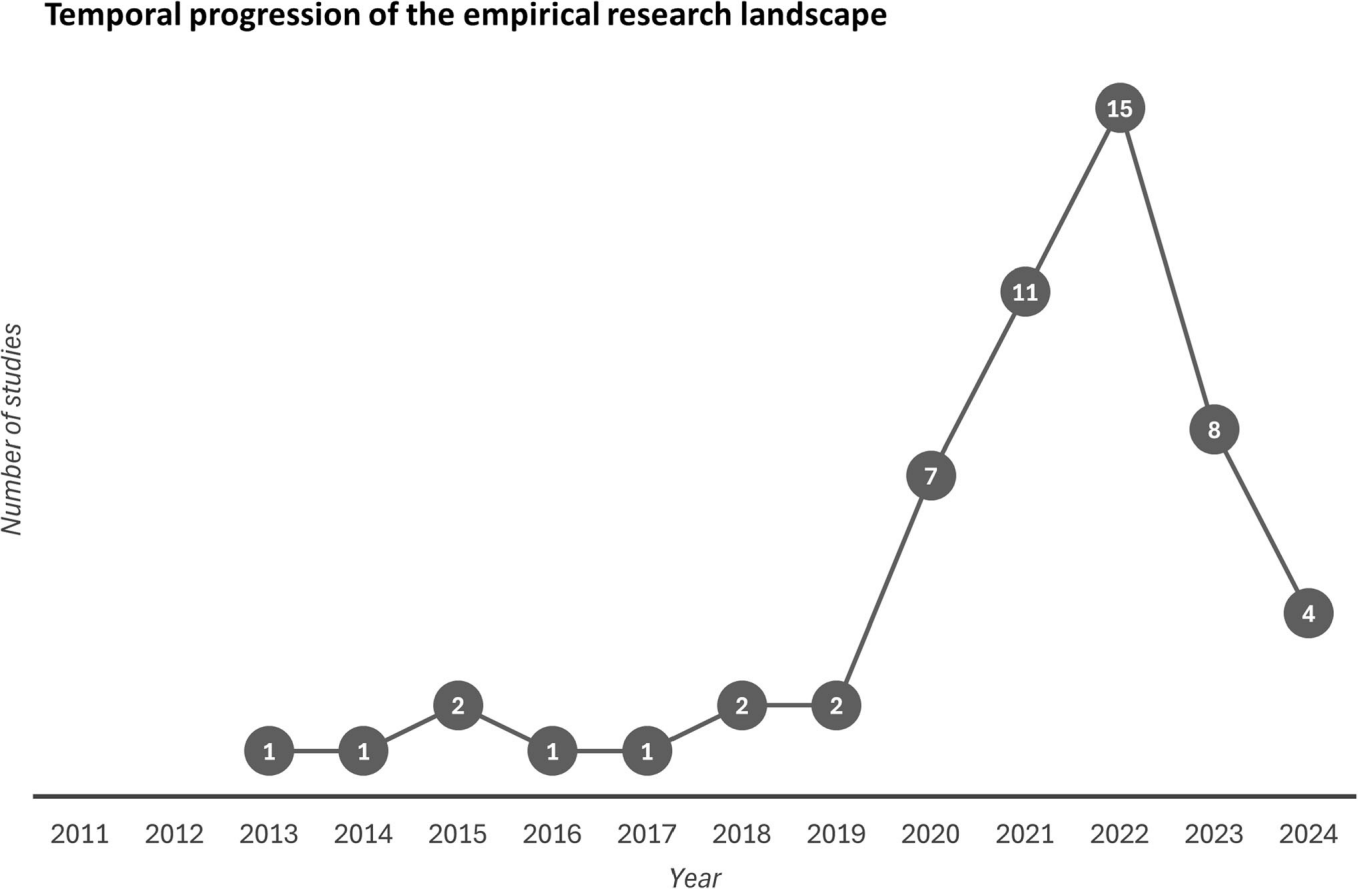
Temporal progression of empirical studies on environmental immobility (*n* = 55) between 2011 (the publication of the Foresight Report) and 2024 (until February 13, 2024, when the literature search was conducted)

Following the Foresight Report’s publication, research on environmental immobility progressed slowly, with researchers describing the topic as under-studied and requiring empirical exploration (Zickgraf 2018). The majority of empirical studies have been conducted since 2020 (Fig. 2). Three broad research themes emerge from the empirical literature: (i) studies which use an open-ended approach to explore *various factors of immobility* in the face of environmental change (Theme 1), (ii) those which focus on the *interactions between environmental change and societal factors* and their impacts on immobility outcomes^14^ (Theme 2), and (iii) those exploring the *nature of immobility processes* by studying subjective experiences of immobility, how immobility decisions are shaped by mobility practices, and questions of agency (Theme 3) (Table 2).

Studies exploring the *various factors of immobility* are primarily conducted in resource-poor settings where livelihoods dependent on natural resources, such as farming and fishing, are disrupted by environmental threats (Milan and Ruano 2014; Paul et al. 2020; Mallick et al. 2022), or where environmental stress is amplified by sociopolitical instability and chronic underdevelopment (Wyngaarden et al. 2023; Santos and Mourato 2024; Upadhyay et al. 2024). Despite adverse conditions, people stay put due to place-based benefits such as livelihood diversification opportunities, access to government aid, and community support (Mallick 2023; Nahin et al. 2023) or, at most, relocate short distances (Paul et al. 2020; Sengupta and Samanta 2022). People also remain in environmentally fragile settings due to apprehensions surrounding migration journeys (Mohammadi and Khanian 2023; Wyngaarden et al. 2023), the lack of financial resources, and reduced economic benefits of migration that effectively ‘trap’ them in place (Milan and Ruano 2014).

A second group of studies aim to understand how environmental threats combine with *certain societal vulnerabilities* in influencing immobility decisions. These studies largely build on Black et al.’s (2011b) conceptual approach on the family of drivers of migration, which shows how migration in hazard-prone settings may not be singularly determined by environmental stress but by an interplay among environmental threats and pre-existing ‘non-environmental’,^15^ i.e. economic, demographic, social and political vulnerabilities. *Economic factors* such as low financial resources and climate change-induced livelihood stress constitute a key concern in the empirical literature. For instance, research explores how poverty and related social inequalities can create mobility constraints in disaster-prone settings at present (Thiede and Brown 2013; Nawrotzki and DeWaard 2018; Otsuyama et al. 2021) or under future climate scenarios (Benveniste et al. 2022). Studies also address how people cope with climate change-induced livelihood stress through a mixed household strategy of mobility and immobility (Khan et al. 2024) and how the diversification of livelihood strategies beyond climate-sensitive sectors equips people to stay and adapt to climatic stress (Biswas and Mallick 2021; Mallick et al. 2020, 2023). Relatedly, research also explores how *political interventions* can enable people to remain in place by promoting the transnational expansion of fragile livelihoods facing climatic change-induced resource depletion (Zickgraf 2019).

Studies examining the *sociopsychological factors* of environmental immobility address ‘negative’ attachments to place, resulting in involuntary immobility, where people may be ‘psychologically trapped’ due to familial obligations (Adams 2016; Ayeb-Karlsson and Uy 2022), well-being losses, and fears of social punishment in migration destination areas (Ayeb-Karlsson et al. 2020; Ayeb-Karlsson 2020d). Studies also uncover cultural motivations that keep people voluntarily in place through ‘positive’ place-based attachments, such as spiritual and emotional attachments to ancestral land and kinship ties (Blondin 2021; Rabbani et al. 2022; Yee et al. 2022a, 2022b; Mavhura 2023). Furthermore, research traces how intergenerational cultures of not evacuating during disaster events and historical knowledge systems of coping with risks influence staying decisions (Klopfer 2017; Haynes et al. 2018; Baer et al. 2019; Wiegel et al. 2021).

Studies examining various *demographic factors,* including life-course transitions, gender identities, and physical (dis-)ability, aim to understand the immobility processes of socially disadvantaged groups. These explore how societal norms surrounding people’s (working) age shape immobility processes in vulnerable households (Makanju and Uriri 2021; Van Praag 2021), the impacts of male outmigration on women ‘left behind’ in high-risk settings (Bhatta et al. 2015; Ahmed and Eklund 2021; Chumky et al. 2023), women’s unequal evacuation opportunities during disasters (Ayeb-Karlsson 2020a, c), their limited access to migration opportunities in the face of reduced livelihoods (Van Praag 2022; Ou-Salah et al. 2024), and the plurality of gendered experiences of immobility practices (Alam and Khalil 2022; Tripathy Furlong et al. 2022; Boas et al. 2023). Research also examines how low mobility potential due to gender hierarchies and inadequate physical competence for undertaking arduous journeys in disaster settings can ‘trap’ people in place (Blondin 2020). Finally, a collection of studies explores instances where disaster-induced *disruptions of material infrastructure*, i.e. roads and transportation systems, lead to immobility outcomes in geographically remote communities which depend on the mobility of people and essential staples for their sustenance (Cook and Butz 2015; Blondin 2020, 2022). In this case, the immobility faced is more material due to impassable routes following disaster events; however, societal norms surrounding gender roles may intersect with physical impassability to produce renewed vulnerabilities.

A third group of studies explore the context-specific *nature of immobility processes* and how people experience these. Scholars explore *subjective narratives* surrounding immobility experiences, for instance, how stayers negotiate societal gender norms (Tripathy Furlong et al. 2022), changing place relations and identities in depopulated regions (Rai 2022), and well-being pressures in the face of slow-onset environmental change (Ayeb-Karlsson and Uy 2022). Studies also explore to what extent women and elderly populations are able to exercise *agency* in their migration and staying decisions, given prevailing societal hierarchies and unequal access to resources (Tripathy Furlong et al. 2022; Boas et al. 2023; Upadhyay et al. 2024). Additionally, studies examining the *interrelations* between immobility and mobility processes show how seasonal and short-term mobility practices help sustain staying aspirations in regions where essential social services and livelihoods have declined under adverse environmental impacts (Cook and Butz 2015; Zickgraf 2019, 2022; Piggott-McKellar and McMichael 2021; Khan et al. 2024). Studies also show how normative discourses surrounding migration practices can fundamentally shape immobility processes (Wyngaarden et al. 2023).

**Fig. 3:**
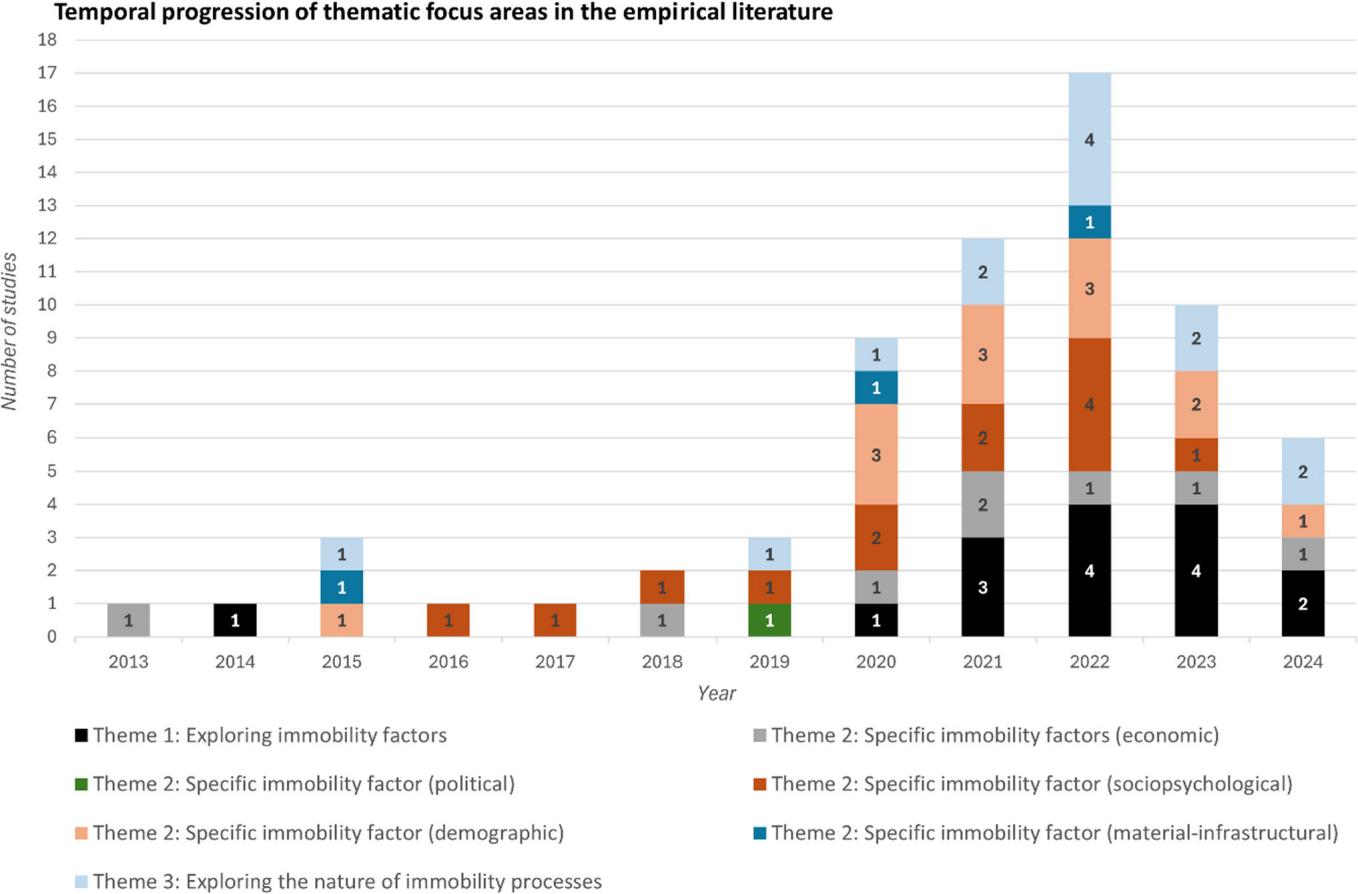
Temporal progression of empirical studies based on thematic focus areas. 11 studies were categorised under two thematic areas (see Table 2), therefore the total number of articles depicted here is 66 (i.e. 11 more than the total number of studies include in the review)

The thematic distribution of articles shows that there has been a predominant focus on understanding *why* people stay put in the face of environmental risks, i.e. the factors of environmental immobility processes. For instance, since 2020, there has been a rise in exploring why people stay put in environmentally fragile locations (Theme 1) (14 studies) and understanding how sociopsychological (9 studies) and demographic (12 studies) factors (in Theme 2) impact immobility decision-making (Fig. 3; Table 2).^16^ In studying how environmental change shapes people’s immobility decisions in concert with societal vulnerabilities, these studies address a research gap pointed out by Black and colleagues (2011b, S9) over a decade ago, at a time when ‘very few studies … sought explicitly to understand the effect of environmental change, or indeed environmental drivers, on mobility [or immobility] in the context of the other drivers of decisions’. In comparison, studies exploring the nature of immobility processes, for instance, to address *how* immobility decisions are made in contexts of unequal power relations and their interrelations with mobility practices, are fewer.

**Table 2:** Thematic categorisations of the reviewed articles. Studies classified under more than one thematic category are represented accordingly and counted separately (11 studies were grouped under two thematic areas, including, Cook and Butz 2015; Zickgraf 2019; Blondin 2020; Ayeb-Karlsson 2020c; McMichael et al. 2021; Ayeb-Karlsson and Uy 2022; Tripathy Furlong et al. 2022; Boas et al. 2023; Wyngaarden et al. 2023; Khan et al. 2024; Upadhyay et al. 2024,  thereby bringing up the total number of articles depicted here to 66)

Theme 1 (*n* = 15)	Theme 2 (economic) (*n* = 8)	Theme 2 (political) (*n* = 1)	Theme 2 (sociopsychological) (*n* = 13)	Theme 2 (demographic) (*n* = 13)	Theme 2 (material-infrastructural) (*n* = 3)	Theme 3 (*n* = 13)
Upadhyay et al. (2024)	Khan et al. (2024)	Zickgraf (2019)	Mavhura (2023)	Ou-Salah et al. (2024)	Blondin (2022)	Khan et al. (2024)
Santos and Mourato (2024)	Mallick et al. (2023)		Rabbani et al. (2022)	Chumky et al. (2023)	Blondin (2020)	Upadhyay et al. (2024)
Nahin et al. (2023)	Benveniste et al. (2022)		Ayeb-Karlsson and Uy (2022)	Boas et al. (2023)	Cook and Butz (2015)	Boas et al. (2023)
Mohammadi and Khanian (2023)	Biswas and Mallick (2021)		Yee et al. (2022a)	Tripathy Furlong et al. (2022)		Wyngaarden et al. (2023)
Mallick (2023)	Otsuyama et al. (2021)		Yee et al. (2022b)	Alam and Khalil (2022)		Ayeb-Karlsson and Uy (2022)
Khatun et al. (2022)	Mallick et al. (2020)		Wiegel et al. (2021)	Van Praag (2022)		Rai (2022)
Mallick et al. (2022)	Nawrotzki and DeWaard (2018)		Blondin (2021)	Van Praag (2021)		Zickgraf (2022)
Wyngaarden et al. (2023)	Thiede and Brown (2013)		Ayeb-Karlsson (2020d)	Ahmed and Eklund 2021		Tripathy Furlong et al. (2022)
Ahsan et al. (2022)			Ayeb-Karlsson et al. (2020)	Makanju and Uriri (2021)		McMichael et al. (2021)
Sengupta and Samanta (2022)			Baer et al. (2019)	Blondin (2020)		Piggott-McKellar and McMichael (2021)
Amin et al. (2021)			Haynes et al. (2018)	Ayeb-Karlsson (2020c)		Ayeb-Karlsson (2020c)
Bhusal et al. (2021)			Klopfer (2017)	Ayeb-Karlsson (2020a)		Zickgraf (2019)
McMichael et al. (2021)			Adams (2016)	Bhatta et al. (2015)		Cook and Butz (2015)
Paul et al. (2020)						
Milan and Ruano (2014)

### Theoretical and methodological concerns

Studies draw on theoretical traditions in migration studies and environmental psychology to understand environmental immobility decision-making. Those addressing immobility drivers (i.e. causality-based research questions) use the aspiration-capability framework and conceptual literature on immobility (e.g. Wyngaarden et al. 2023; Ou-Salah et al. 2024) in their work. These approaches (Carling 2002; Carling and Schewel 2018; Schewel 2020) have helped in analytically distinguishing the factors that motivate people to remain put and those that constrain movement. Studies aiming to understand interactions between immobility and mobility processes within households use other approaches from migration theory, including the new economics of labour migration (NELM) (e.g. Khan et al. 2024) and the mobilities paradigm (e.g. Cook and Butz 2015; Blondin 2020, 2022; Zickgraf 2022). NELM (Stark and Bloom 1985) helps examine migration and non-migration practices as mutually supporting strategies for spreading environmentally-induced livelihood risks at the household-level, while the mobilities paradigm (Sheller and Urry 2006) helps uncover the interactions between and the multiplicities of mobility and immobility processes at a wider scale, across individual, household, and societal levels (Wiegel et al. 2019). Studies draw on concepts from environmental psychology, including place attachment, place belongingness and place relations (e.g. Blondin 2021; Rabbani et al. 2022; Yee et al. 2022a; Mavhura 2023) to explore the experiences and motivations of people who voluntarily stay put due to place-based ties. Studies also develop analytical frameworks of immobility decision-making processes (e.g. Ahsan et al. 2022; Khatun et al. 2022; Mallick 2023; Mallick et al. 2023) based on conceptual literature on the multicausality of environmental migration (Black et al. 2011b) and livelihood resilience in the face of climatic stress (Tanner et al. 2014).

**Fig. 4:**
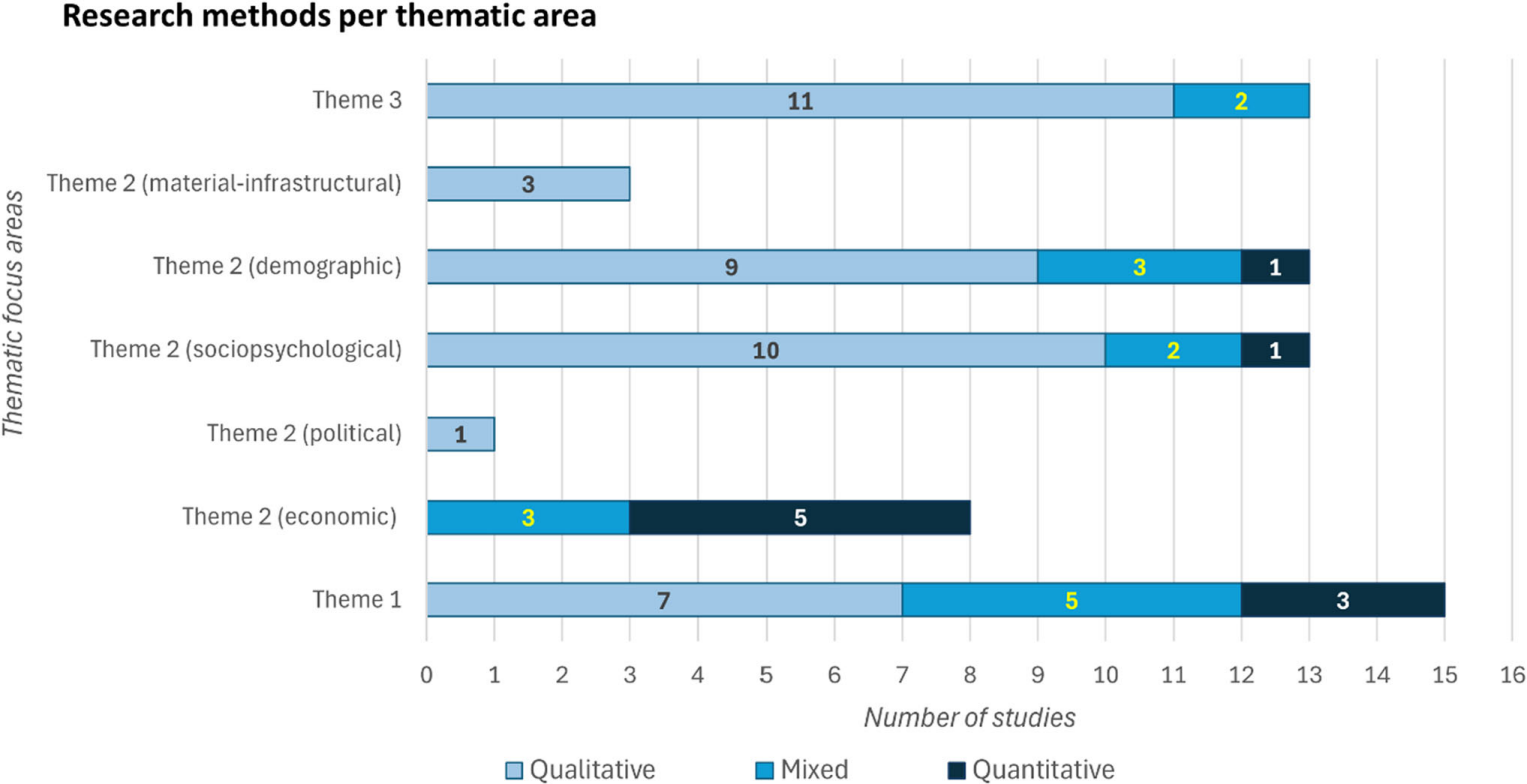
Research methods used based on thematic areas

A majority of studies use qualitative research methods (32), followed by mixed research methods (13), and quantitative methods (10). Qualitative approaches are particularly used in studies exploring the nature of immobility processes (Theme 3), whereas quantitative and mixed designs are used in studies addressing the factors of immobility (Themes 1 and 2), for instance, those examining correlations between various social, economic or demographic factors and immobility outcomes under environmental stress (e.g. Thiede and Brown 2013; Nawrotzki and DeWaard 2018) (Fig. 4). In quantitative studies involving household surveys, the unit of analysis is taken to be the household where the household head is typically interviewed (e.g. Mallick et al. 2022, 2023), which can lead to the invisibilisation of more disadvantaged household members (Zickgraf 2021). In contrast, studies using qualitative approaches take the individual to be the unit of analysis, which helps in better representing the needs and vulnerabilities faced by all household members, particularly in research settings where societal norms may inhibit interactions with female participants (Castillo Betancourt and Zickgraf 2024). In their attempt to accurately represent immobility experiences, some qualitative studies also employ context-sensitive and culturally relevant methods of data collection, such as storytelling session in Bangladesh (Ayeb-Karlsson 2020a) and ‘talanoa’ sessions in Fiji (Yee et al. 2022a, 2022b), which simultaneously help in reducing hierarchies in research relationships.

### Regions studied

**Fig. 5:**
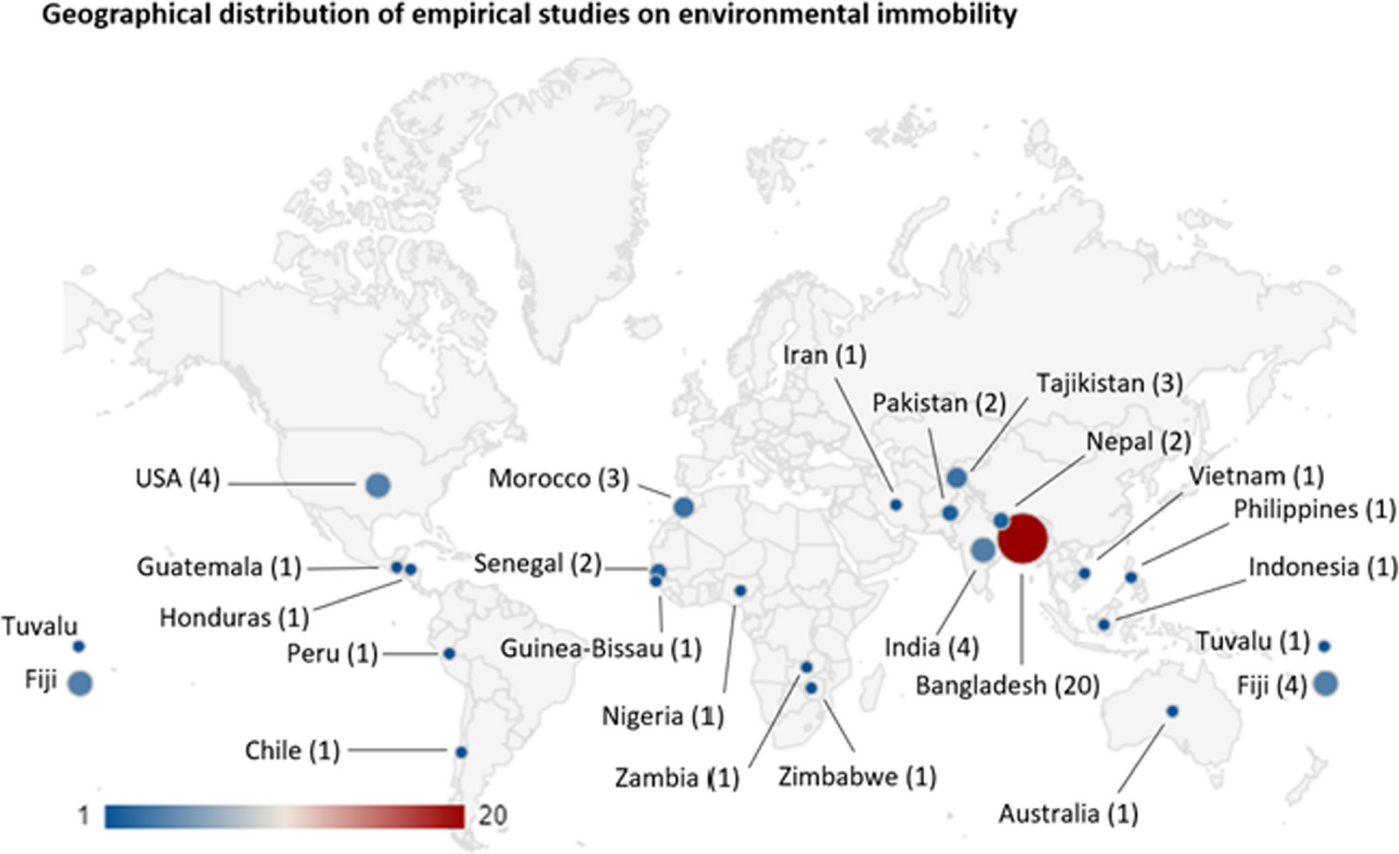
Geographical distribution of empirical studies with the number of publications per country. In the case of studies conducted in multiple countries (e.g. Bhatta et al. 2015; Zickgraf 2019; McMichael et al. 2021), each study country was counted separately

The geographical distribution of studies on environmental immobility (Fig. 5) is disproportionately concentrated in a few countries and regions, as has been the case in the broader environmental migration research field (Piguet et al. 2018). A large number of studies have been conducted in Bangladesh (20 studies), followed by Fiji (4 studies), India (4 studies), USA (4 studies), Morocco (3 studies), Tajikistan (3 studies), Nepal (2 studies), Pakistan (2 studies), and Senegal (2 studies). Fourteen countries located in central and southern America, west and southern Africa, west and East Asia, and Oceania have merited only one study. The predominance of empirical research on immobility in Bangladesh may be explained by the severe climatic risks and developmental deficits that the country faces (Piguet et al. 2018), and high population density along coastlines despite exposure to coastal flooding and rising sea levels (Bell et al. 2021).^17^ However, this leaves other regions facing high climatic risks, such as those in sub-Saharan Africa, Central America and other small island states, outside the academic radar of immobility scholarship. Diversity of geographical settings in studies is important not only because it accounts for regions facing high levels of risks but also because regional differences in societal norms and local perceptions of migration and staying practices can have different implications for resulting immobility and mobility processes.^18^

In terms of empirical settings, studies were largely conducted in rural areas in coastal, e.g. (McMichael et al. 2021; Mallick et al. 2023), mountainous (Blondin 2020; Upadhyay et al. 2024), and riparian seatings (e.g. Mavhura 2023; Nahin et al. 2023). The over-concentration of research in rural areas may be explained by the vulnerability of agrarian livelihoods to environmental threats and resulting socioeconomic constraints on movement in instances where non-farming income opportunities are scarce. The precarity of agrarian livelihoods variously affects immobility and mobility decisions, for instance, by intersecting with local gender roles and triggering male outmigration, while women, children, and the elderly typically stay behind (e.g. Tripathy Furlong et al. 2022; Upadhyay et al. 2024). Studies in urban areas are relatively under-researched compared to rural settings (Ayeb-Karlsson 2020d) and explore a range of mobility constraints such as poverty, adverse health conditions, poor living conditions in urban informal settlements (Ayeb-Karlsson et al. 2020; Ayeb-Karlsson 2020d), and social marginalisation (Thiede and Brown 2013) that prevent people from migrating away from high-risk settings.

### The interplay of the environmental and societal contexts

In the empirical literature, people were found to be variously impacted by environmental stressors (Table 3). In coastal and island settings,^19^ people face a range of sudden-onset hazards such as hurricanes, cyclonic storms, storm surges, and coastal flooding and slow-onset environmental changes including sea-level rise, land and water degradation, and biodiversity losses. These lead to infrastructural losses and population displacements in urban regions (e.g. Baer et al. 2019; Otsuyama et al. 2021), and diminished livelihoods and well-being losses in rural areas, triggering labour mobilities from affected households (e.g. Ahmed and Eklund 2021; Santos and Mourato 2024). People located in flood plains in riparian and deltaic settings^20^ are similarly confronted with livelihood losses, triggering outmigration of partial households (typically male members) in search of alternative income sources (e.g. Bhatta et al. 2015). In inland settings with dry climates,^21^ slow-onset changes including droughts, desertification and changes in temperature and precipitation patterns result in water scarcity and the unviability of agricultural livelihoods (Mohammadi and Khanian 2023; Ou-Salah et al. 2024). In mountain settings,^22^ people are confronted with multiple sudden-onset hazards including landslides, avalanches, glacial outbursts, and flooding, and slow-onset changes including changing temperature and precipitation patterns, glacial retreat, and drought. These result in resource scarcity, diminished livelihoods (Bhusal et al. 2021; Upadhyay et al. 2024), and disrupted mobility infrastructure which reduces access to basic services and staple goods in remote regions (Cook and Butz 2015; Blondin 2020).

**Table 3:** Summary of reviewed articles (*n* = 55)

No	Articles (*n* = 55)	Research aim	Research approach	Study region	Environmental factors and events	Environmental impacts	Societal context and vulnerabilities	Immobility outcomes	Research theme(s) categorisation
1	Khan et al. (2024)	Climate change impacts on mountain livelihoods and resulting (im)mobility patterns and interrelations	Mixed	Rural mountain communities in the Eastern Hindu Kush region, Pakistan	Snow avalanche, glacial outburst, landslide, rockfall, river erosion, land subsidence, debris flow	Livelihood stress (land degradation, decline in crop productivity and livestock farming), population displacement, labour migration	Low economic resources and assets, subsistence livelihoods	Involuntary	Theme 2 (economic); Theme 3
2	Upadhyay et al. (2024)	Immobility experiences, drivers, and interrelations with mobility processes in highly mobile mountain communities	Qualitative	Rural mountain communities in the Himalayan region, India	Temperature and precipitation anomalies, drought	Livelihood stress (land degradation, decline in traditional crop yield and native agrobiodiversity), gradual depopulation	Developmental deficit, subsistence livelihoods, place attachment, community support, remittances, age, gender	Voluntary and involuntary (Both)	Theme 1; Theme 3
3	Ou-Salah et al. (2024)	Gendered impacts of slow-onset environmental change; migration barriers and aspirations of immobile women	Qualitative	Rural community in Morocco	Aridity, drought, water scarcity	Livelihood stress (decline in agricultural and livestock farming)	Gender (women employed in the low-paying agrarian sector)	Uncategorised	Theme 2 (demographic)
4	Santos and Mourato (2024)	Climate change perceptions and factors of non-migration despite environmental risks, poverty and inequalities	Qualitative	Rural and urban coastal communities in Guinea Bissau	Sea-level rise, flooding, erosion, storms, drought, temperature and precipitation variations	Livelihood stress (decline in livestock farming, agriculture and fishing)	Dysfunctional governance, developmental deficit, economic inequality, subsistence livelihoods, self-reliance, solidarity	Voluntary	Theme 1
5	Boas et al. (2023)	Plural and situated exploration of gender, environmental change, and (im)mobility interrelations	Qualitative	Rural coastal communities in Bangladesh	Cyclones, flood, river erosion, sea-level rise	Livelihood stress, decline in habitability, seasonal labour mobilities and relocation of entire families	Gender (patriarchal norms constraining women’s mobility)	Both	Theme 2 (demographic); Theme 3
6	Chumky et al. (2023)	Impacts of male outmigration and climate adaptation strategies of women ‘left behind’ in migrant households	Mixed	Rural coastal communities in Bangladesh	Salinity intrusion	Livelihood stress (agricultural losses), water scarcity, adverse health impacts, male labour outmigration	Gender (patriarchal norms constraining women’s mobility)	‘Left behind’	Theme 2 (demographic)
7	Wyngaarden et al. (2023)	Agentic choices of immobility and (im)mobility interrelations in a context of high outmigration, economic and social challenges, and climate change-induced livelihood stress	Qualitative	Rural communities in Honduras	Unpredictable rainy season, droughts	Livelihood stress (decline in agricultural yield), food insecurity	Poverty, economic inequality, social instability, subsistence livelihoods, moral discourses surrounding mobility and immobility	Voluntary	Theme 1; Theme 3
8	Nahin et al. (2023)	Environmental immobility drivers and associated adaptation strategies	Mixed	Rural riverine communities in Bangladesh	Riverbank erosion, flooding	Livelihood stress (damage to crops and livestock) and loss of key infrastructure	‘Cooperative’ and ‘obstructive’ factors facilitating immobility (e.g. government and community support, access to flood shelters) or obstructing mobility (e.g. poverty, illiteracy, lack of marketable skills)	Voluntary	Theme 1
9	Mavhura (2023)	Place attachment influencing resistance to relocation and immobility despite disaster risk	Qualitative	Rural riparian communities in Zimbabwe	Floods, dry spells and drought	Livelihood stress (loss of crops and livestock), infrastructural damage, adverse health impacts	Place attachment to land, forest resources, and community	Voluntary	Theme 2 (sociopsychological)
10	Mohammadi and Khanian (2023)	Immobility factors in the context of climate change impacts and a culture of high outmigration	Qualitative	Rural communities in Iran	Drought	Livelihood stress (agricultural losses) and water scarcity	Livelihood precarity and poverty, uncertainty surrounding migration outcomes, social network, place attachment	Both	Theme 1
11	Mallick (2023)	Factors of environmental non-migration	Quantitative	Rural coastal communities in Bangladesh	Cyclones	Livelihood stress and population displacement	Social network, access to capital, land ownership and economic opportunities, community cohesion	Voluntary	Theme 1
12	Mallick et al. (2023)	Livelihood resilience as a factor of voluntary environmental non-migration	Mixed	Rural coastal communities in Bangladesh	Cyclones	Livelihood stress and population displacement	Livelihood resilience	Voluntary	Theme 2 (economic)
13	Khatun et al. (2022)	Factors of voluntary environmental non-migration	Quantitative	Rural coastal communities in Bangladesh	Tropical cyclones, flooding, riverbank erosion, heavy rainfall	Livelihood stress (loss of agricultural yield), water scarcity, loss of well-being, social conflicts over resources	Access to basic needs and natural resources, community support, place attachment	Voluntary	Theme 1
14	Rabbani et al. (2022)	Influence of place relations on environmental non-migration decision-making	Qualitative	Rural coastal communities in Bangladesh	Cyclones, storm surges, salinity intrusion, coastal erosion	Livelihood stress (crop losses, decline in livestock and native fish species), food insecurity, developmental crises	Place relations: place-based livelihoods, place obduracy, risk perceptions, sociostructural constraints of mobility	Both	Theme 2 (sociopsychological)
15	Mallick et al. 2022	Factors of voluntary environmental non-migration	Quantitative	Rural coastal communities in Bangladesh	Salinity intrusion, siltation	Livelihood stress (agricultural losses), water scarcity	Land ownership, social network, economic strength	Voluntary	Theme 1
16	Ayeb-Karlsson and Uy (2022)	People’s experiences and narratives concerning slow-onset environmental change and (im)mobility outcomes	Qualitative	Island communities in the Philippines	Land and water degradation, shifting rainfall and temperature patterns, drought and dry spells, biodiversity losses	Livelihood stress, infrastructural damage, loss of ecosystem services, poverty, outmigration	Lack of social connections and financial resources, caregiving responsibilities	Both; ‘left behind’	Theme 2 (sociopsychological); Theme 3
17	Zickgraf (2022)	Capture the fluidity and relationality of environmental mobility and immobility processes	Qualitative	Urban, coastal fishing community in Senegal	Coastal erosion, sea-level rise, flooding, soil salinisation, storm surges	Livelihood stress (decline in fish stocks)	Place-based rootedness of identities and skills, livelihood diversification through international labour mobilities	Voluntary	Theme 3
18	Ahsan et al. (2022)	Factors of voluntary environmental non-migration	Mixed	Rural coastal communities in Bangladesh	Tropical cyclones, coastal flooding, salinity intrusion	Livelihood stress (agricultural losses)	Place-based socioeconomic and psychosocial advantages, access to natural resources, community support, lower disaster risk perception	Voluntary	Theme 1
19	Benveniste et al. (2022)	Modelling declining international mobility due to resource deprivation under future climate scenarios	Quantitative	Global dataset	Future climate scenarios	Resource deprivation	Poverty	Involuntary (‘Resource-constrained immobility’)	Theme 2 (economic)
20	Yee et al. (2022b)	Indigenous value system as a factor of voluntary immobility and resistance to relocation	Qualitative	Rural island communities in Fiji	Sea-level rise, flooding, saltwater intrusion	Declining habitability, food insecurity (declining fish stocks, crops and vegetable yields), infrastructure damage	Indigenous Fijian value system; place attachment	Voluntary	Theme 2 (sociopsychological)
21	Blondin (2022)	Disaster-induced mobility disruptions and resulting mobility challenges	Qualitative	Rural mountain communities in the Bartang Valley, Tajikistan	Avalanches, rockslides, floods, mudslides	Disruption of mobility infrastructure; reduced, dangerous, and arduous mobilities; diminished habitability	Poor mobility infrastructure (lack of public transportation and low motorisation rate),	Involuntary	Theme 2 (material-infrastructural)
22	Tripathy Furlong et al. (2022)	Gendered emotions, sociocultural contexts and (im)mobility experiences	Qualitative	Rural coastal communities in Bangladesh	Cyclone, riverbank erosion, flooding, salinity intrusion	Livelihood stress, male labour outmigration	Gender: gendered experiences of home and belongingness, identity constructions, environmental risk perception and management	Voluntary	Theme 2 (demographic); Theme 3
23	Rai (2022)	Subjective experiences of people staying put in a context of environmental change and depopulation	Qualitative	Rural mountain communities in the Himalayan region, India	Declining natural resources, erratic precipitation, advancing cropping season, flash floods, drying of perennial streams	Food insecurity, land abandonment and depopulation	Lack of development initiatives	‘Left behind’	Theme 3
24	Sengupta and Samanta (2022)	Factors of environmental immobility	Mixed	Rural coastal communities in India	Cyclones, storm surges, coastal erosion	Erosion and shrinkage of agricultural and residential land, livelihood disruptions and land use changes	Livelihood diversification and circular migration, government support, place attachment, social capital	Voluntary	Theme 1
25	Yee et al. (2022a)	Place belongingness as a factor of voluntary immobility and resistance to relocation	Qualitative	Rural island communities in Fiji	Sea-level rise and associated coastal erosion, storm surges, salinity intrusion	Decline in agricultural yield and fish stocks, food insecurity, submergence of ancient burial sites, and loss of mental well-being	Place belongingness: autobiographical, ancestral, relational, cultural, economic, legal connections to place	Voluntary	Theme 2 (sociopsychological)
26	Alam and Khalil (2022)	Impacts of environmental change-induced male labour migration on immobile women	Qualitative	Rural coastal communities in Bangladesh	Cyclone, storm surges, coastal flooding, salinity	Livelihood stress leading to labour migration	Gender: Patriarchal norms constraining women’s mobility, unequal access to resources	Uncategorised	Theme 2 (demographic)
27	Van Praag (2022)	Gendered impacts of environmental change, women’s migration aspirations, abilities and immobility outcomes	Qualitative	Urban communities in Morocco	Changes in precipitation and temperature patterns, drought, desertification, water scarcity	Women’s livelihood stress owing to the feminisation of subsistence agriculture	Gender: Feminisation of subsistence agriculture, gendered caregiving responsibilities	Uncategorised	Theme 2 (demographic)
28	Amin et al. (2021)	Factors of immobility in a disaster-prone setting	Qualitative	Coastal fishing community in Indonesia	Flooding, land subsidence	Disruption of coastal life and livelihoods	Place attachment, family ties, social ties, occupational ties	Voluntary	Theme 1
29	Bhusal et al. (2021)	Factors of non-migration in landslide-prone mountain communities	Qualitative	Rural mountain communities in the Himalayan region, Nepal	Flooding, landslides, forest fires, windstorms, hailstorms, drought	Livelihood stress (agricultural losses)	Place attachment, place confidence, social capital, livelihood diversification	Voluntary	Theme 1
30	Biswas and Mallick (2021)	Livelihood diversification as a factor of non-migration despite environmental risks	Quantitative	Rural coastal communities in Bangladesh	Cyclones, flooding, tidal surges, heavy rainfall, riverbank erosion	Livelihood stress, occupational disruptions and land use changes	Livelihood diversification	Voluntary	Theme 2 (economic)
31	Wiegel et al. (2021)	People’s ontological security and subjective risk perception as a factor of voluntary immobility and resistance to relocation	Qualitative	Rural mountain community in Chile	Mudslide event, 2017	Infrastructural damage and loss of lives	Subjective risk perceptions	Voluntary	Theme 2 (sociopsychological)
32	Ahmed and Eklund (2021)	Impacts of climate-induced male outmigration on ‘left-behind’ women	Qualitative	Rural coastal communities in Bangladesh	Sea-level rise, tropical cyclones, storm surge, coastal flooding, coastal erosion, variations in rainfall patterns	Livelihood stress, food insecurity, male seasonal labour migration, impacts on left-behind women	Gender: Women’s socioeconomic vulnerabilities, exposure to risks, mobility constraints	‘Left behind’	Theme 2 (demographic)
33	McMichael et al. (2021)	People’s experiences of immobility and translocality in low-lying island communities; factors of immobility despite coastal hazards	Qualitative	Rural island communities in Fiji and Tuvalu	Sea-level rise, coastal erosion, tidal surges, flooding, saltwater intrusion	Livelihood stress and food insecurity (decline in subsistence crops), loss of land and ancient burial sites	Place attachment, cultural rootedness, place-based livelihood opportunities	Voluntary	Theme 1; Theme 3
34	Otsuyama et al. (2021)	Immobility outcomes based on access to economic resources and housing insurance	Quantitative	Urban coastal communities in the USA	Hurricane Irma, 2017	Infrastructural damage to housing and property	Household economic resources and housing insurance	Both	Theme 2 (economic)
35	Blondin (2021)	Place attachment as a factor of voluntary immobility despite disaster risks	Qualitative	Rural mountain communities in Tajikistan	Avalanches, rockslides, floods, mudslides	Threats to life, population displacement, disruption of roads and transportation infrastructure	Place attachment: Place identity and dependence, social bonding, biophysical bonding	Voluntary	Theme 2 (sociopsychological)
36	Van Praag (2021)	Life course as a factor of immobility in the context of slow-onset environmental change	Qualitative	Urban communities in Morocco	Precipitation and temperature changes, drought, desertification, water scarcity	Livelihood stress (losses incurred in rainfed agriculture)	Life course	Uncategorised	Theme 2 (demographic)
37	Makanju and Uriri (2021)	Immobility decision-making among aged residents at environmental risk	Quantitative	Rural communities in Nigeria	Temperature and precipitation anomalies, drought	Livelihood stress (agriculture)	Age, household resilience	Involuntary (‘Trapped populations’)	Theme 2 (demographic)
38	Piggott-McKellar and McMichael (2021)	Interrelations between climate-related immobility and relocation responses	Qualitative	Rural island communities in Fiji	Sea-level rise, coastal erosion, tidal inundation, storm surges	Declining habitability, livelihood and food insecurity	Adaptation measures, community consensus, cooperation and leadership, land rights and tenure, institutional support	Both	Theme 3
39	Mallick et al. (2020)	Influence of varying livelihood conditions in different socioecological systems on future migration and non-migration decisions	Mixed	Rural coastal communities in Bangladesh	Flood, erratic rainfall, temperature variations, drought, salinity	Livelihood stress (Reduced agricultural productivity)	Sustainable livelihoods, livelihood diversification	Voluntary	Theme 2 (economic)
40	Blondin (2020)	Low motility (mobility potential) as a factor of involuntary immobility	Qualitative	Rural mountain communities in the Bartang Valley, Tajikistan	Avalanches, rockslides, floods, landslides	Disruption of roads and transportation infrastructure	Low mobility potential due to low access, competencies and appropriation of mobility	Involuntary	Theme 2 (material-infrastructural); Theme 2 (demographic)
41	Ayeb-Karlsson (2020d)	Psychological and social connotations of ‘trapped populations’	Qualitative	Urban informal settlement in Bangladesh	Bhola Cyclone, 1970, riverbank erosion	Loss of land and population displacement	Psychological well-being losses	Involuntary (‘Trapped populations’)	Theme 2 (sociopsychological)
42	Ayeb-Karlsson (2020a)	Gendered narratives concerning women’s non-evacuation during disaster events	Mixed	Rural coastal communities in Bangladesh	Cyclones	Loss of mental and physical well-being	Gender: Women’s vulnerabilities and mobility constraints	Involuntary (‘Trapped populations’)	Theme 2 (demographic); Theme 3
43	Ayeb-Karlsson (2020c)	Women’s immobility during disasters due to gendered conceptions of safe spaces, gendered knowledge systems, and gendered experiences of fear and trauma	Qualitative	Rural coastal communities in Bangladesh	Cyclones	Loss of mental and physical well-being	Gender: Women’s vulnerabilities and mobility constraints	Involuntary (‘Trapped populations’)	Theme 2 (demographic)
44	Ayeb-Karlsson et al. (2020)	Climate-induced immobility in an urban informal settlement and associated well-being losses	Mixed	Urban informal settlement in Bangladesh	Bhola Cyclone, 1970, riverbank erosion	Loss of land and population displacement	Psychological well-being losses	Involuntary (‘Trapped populations’)	Theme 2 (sociopsychological)
45	Paul et al. (2020)	Factors of voluntary immobility of people displaced by riverbank erosion	Mixed	Deltaic community in the Meghna River estuary in Bangladesh	Riverbank erosion	Loss of agricultural produce, infrastructural damages, loss of ancient burial sites, population displacement, decline in economic opportunities	Social, cultural, financial, political, human, natural, built capital and place attachment	Voluntary	Theme 1
46	Baer et al. (2019)	Influence of regional cultural values and beliefs on hurricane risk perception and non-evacuation outcomes	Qualitative	Island city in the USA	Hurricane Ike, 2008	Inundation and damage of infrastructure, depopulation	Cultural values influencing (low) risk perception	Both	Theme 2 (sociopsychological)
47	Zickgraf (2019)	Political interventions facilitating the long-term non-migration abilities of people	Qualitative	Urban, coastal fishing community (Senegal); rural riverine communities (Vietnam)	Sea-level rise, coastal and riverbank erosion, storm surges, flooding, soil salinisation, rainfall variability, mudslides	Livelihood stress (low agricultural productivity, reduced fish stocks)	Political interventions: International labour mobility agreement (Senegal) and internal short-distance relocation (Vietnam)	Uncategorised	Theme 2 (political); Theme 3
48	Nawrotzki and DeWaard (2018)	The economic characteristics of migrant-sending places and access to migrant networks as factors influencing immobility outcomes	Quantitative	Zambian districts	Heat waves, drought	Livelihood stress	Fragile and subsistence livelihoods, migration networks	Involuntary (‘Trapped populations’)	Theme 2 (economic)
49	Haynes et al. (2018)	Disaster non-evacuation motivated by intergenerational culture of sheltering-in-place	Mixed	Urban coastal community in Australia	Cyclone Debbie, 2017 and associated flooding	Infrastructural damage and fatality	Intergenerational culture of sheltering-in-place during disasters	Voluntary	Theme 2 (sociopsychological)
50	Klopfer (2017)	Exploring the historical and sociopsychological roots of the narrative of ‘choosing to stay’ during Hurricane Katrina	Qualitative	Urban coastal community in the USA	Hurricane Katrina, 2005	Infrastructural damage, loss of lives, population displacement	Historical roots of disaster immobility, place-based knowledge, narratives on the right to the city	Voluntary	Theme 2 (sociopsychological)
51	Adams (2016)	Sociopsychological and affective factors of involuntary immobility	Quantitative	Rural mountain communities in the Rimac River valley in the Andes, Peru	Temperature and precipitation anomalies, loss of ice in higher altitudes, changes in the predictability of seasons	Livelihood stress, water stress, adverse health impacts, infrastructural damage and mobility constraints	Resource barriers, ‘negative’ place attachment	Involuntary (‘Trapped populations’)	Theme 2 (sociopsychological)
52	Bhatta et al. (2015)	Impacts of extreme weather events and male outmigration on women staying put	Mixed	Rural agricultural communities in India, Bangladesh, Nepal	Drought, flooding, cyclones, salinity intrusion, increasing temperatures and erratic rainfall patterns, cold spells	Livelihood stress, male outmigration	Gender: Women’s vulnerabilities and mobility constraints	‘Left behind’	Theme 2 (demographic)
53	Cook and Butz (2015)	Dialectical constitution of mobility and immobility in a disaster setting	Qualitative	Rural mountain communities in Pakistan	Attabad Landslide, 2010	Road and infrastructural damage leading to mobility disruptions; decline in income sources, access to healthcare, education, kinship ties	Geographical remoteness, lack of infrastructure, remobilisation strategies	Involuntary (‘strandedness’)	Theme 2 (material-infrastructural); Theme 3
54	Milan and Ruano (2014)	Factors ‘trapping’ people in place	Mixed	Rural mountain communities in Guatemala	Changes in rainfall patterns, drought, dry spells, and flooding	Livelihood stress, food insecurity, declined profitability of alternative in-situ livelihoods	Declining labour migration opportunities	Involuntary (‘Trapped populations’)	Theme 1
55	Thiede and Brown (2013)	The influence of race and socioeconomic status on hurricane non-evacuation behaviour	Quantitative	Urban coastal community in the USA	Hurricane Katrina, 2005	Infrastructural damage, loss of lives, population displacement	Race, education, information attainment, local embeddedness/social ties	Both	Theme 2 (economic)

Environmental impacts and resultant immobility processes were found to vary across population groups largely based on social and economic differentiations. For instance, studies reveal that people from lower socioeconomic backgrounds (e.g. Nawrotzki and DeWaard 2018; Mohammadi and Khanian 2023), racial minorities (e.g. Thiede and Brown 2013), those engaged in climate-sensitive occupations (e.g. Mallick et al. 2020; Rabbani et al. 2022), women (e.g. Bhatta et al. 2015; Ayeb-Karlsson 2020c), the aged (e.g. Makanju and Uriri 2021; Van Praag 2021), and those with low physical ability (Blondin 2020, 2022) are most vulnerable to the adverse impacts of environmental change and face multiple mobility constraints, leading to involuntary immobility outcomes. In contrast, people engaged in livelihoods not directly dependent on environmental conditions, from higher income backgrounds, and those with access to education and financial resources possess higher decision-making abilities concerning their responses to environmental change (Thiede and Brown 2013; Biswas and Mallick 2021). Similarly, societal norms surrounding gender hierarchies and divisions of labour accord greater mobility potential to men compared to women (Ayeb-Karlsson 2020a, c; Van Praag 2022).

However, the interplay between environmental change and social vulnerabilities may not always result in linear and homogenous implications for immobility outcomes. Studies point to instances where people willingly stay put in high-risk settings and experience significant well-being gains despite being characterised by economic and social vulnerabilities. For instance, while poverty and livelihood stress typically prevent people from migrating away from risks, thereby leading to involuntary immobility (Mohammadi and Khanian 2023; Khan et al. 2024), they may also stay put willingly due to access to abundant natural resources offering food security and sustenance (McMichael et al. 2021) and opportunities to diversify their income sources locally (Biswas and Mallick 2021; Mallick et al. 2020, 2023).

Demographic factors such as gender hierarchies were also found to influence immobility outcomes differently across settings. In some study regions, patriarchal norms inhibit women’s mobility during disasters (Ayeb-Karlsson 2020a, c) and diminish their abilities to undertake short-term labour mobilities when place-based livelihoods decline (Ou-Salah et al. 2024). These studies find women to be ‘trapped’ (Ayeb-Karlsson 2020a, c; Van Praag 2022; Ou-Salah et al. 2024) or having been ‘left behind’ by male migrant household members (Bhatta et al. 2015; Ahmed and Eklund 2021; Chumky et al. 2023) in hazard-prone regions. However, other studies find contrasting impacts of environmental change on women’s immobility experiences. In the absence of migrant male household members, women stayers were found to experience greater financial and social freedoms including increased decision-making power in undertaking local mobilities, developing women-led livelihood interventions, and taking charge of household resources that were previously managed by men (Alam and Khalil 2022; Tripathy Furlong et al. 2022; Boas et al. 2023). In highlighting the heterogeneity of immobility experiences by women stayers, these studies challenge the stereotypical understanding of women as powerless actors in immobility processes.

### Characterisation of immobility processes

As in wider migration studies research (Carling 2002; Carling and Schewel 2018), immobility outcomes in environmental migration research have been characterised in terms of ‘voluntary’ and ‘involuntary’ immobility^23^ to distinguish between people unable to exit high-risk settings due to a lack of migration capabilities and those who willingly remain in place (Table 3). Under involuntary immobility processes, studies also include the immobility experiences of ‘left-behind’ people comprising women staying behind to support in-situ livelihoods while men outmigrate (Bhatta et al. 2015; Ahmed and Eklund 2021; Chumky et al. 2023) and elderly populations ‘left behind’ in communities facing climate change-induced developmental challenges (Rai 2022).^24^

Questioning the dichotomisation of environmental immobility outcomes as either voluntary or involuntary, recent studies argue that agency in immobility decisions falls along a continuum of voluntary and involuntary outcomes (Boas et al. 2023; Upadhyay et al. 2024). This is because people may *simultaneously indicate the desire to stay put as well as the inability to migrate* from settings marked by environmental stress, e.g. women experiencing mobility constraints due to societal gender norms may simultaneously choose to stay put due to place-based attachments (Ayeb-Karlsson 2020c; Tripathy Furlong et al. 2022; Upadhyay et al. 2024) or Indigenous people choosing to staying put due to place-based cultural ties may at the same time face mobility barriers owing to a lack of access to alternative customary land outside their village (McMichael et al. 2021). Furthermore, studies also reveal that it may be difficult to identify the extent of voluntariness in immobility decisions due to *complex interactions between the individual and structural factors* influencing immobility outcomes. For instance, in settings where the larger community attributes positive moral value to staying in place, individual immobility aspirations may be hard to distinguish from socially derived behaviour (Wyngaarden et al. 2023), thereby bringing into question the extent of choice available to stayers. Similarly, while people staying put due to place-based attachments may seemingly be doing so voluntarily, there may be structural mobility barriers in the form of locally embedded occupational skills (e.g. farming in specific biophysical conditions) that preclude any real choice regarding staying decisions (Rabbani et al. 2022).

### Interrelations with mobility processes

Empirical studies additionally address how immobility processes interact with mobility processes within households and communities. Three aspects emerge from the reviewed literature––First, immobility and mobility constitute *functionally enabling processes* that help people adapt to climate-induced livelihood stress and reduced habitability. For instance, the labour migration of a few household members enables the immobility of others through the provision of remittances to support the household’s in-situ income sources (Zickgraf 2019, 2022; Khan et al. 2024). Similarly, weekly mobilities to urban centres ensure access to basic services and supplementary income sources while continuing to remain put in rural areas facing diminished livelihoods (McMichael et al. 2021). Second, immobility and mobility constitute *spatially and temporally interchangeable processes*. Studies suggest that immobile actors are not spatially static in regions exposed to environmental stress but instead engage in a range of small-scale and circular mobilities such as internal relocation initiatives, everyday mobilities and local commercial activities (Zickgraf 2022; Boas et al. 2023) as well as ‘remobilisation’ efforts to overcome disaster-induced disruptions of key roads and transportation services (Zickgraf 2019, 2022). Similarly, instead of being fixed categories, immobility and mobility processes were found to be interchangeable over time, e.g. those practising mobility at a certain point of time by relocating short distances or practising circular labour mobilities may subsequently practise immobility (Zickgraf 2019, 2022).

Third, immobility and mobility constitute *experientially overlapping processes*. Two kinds of experiential overlaps emerge in the empirical literature––in the first instance, narratives of people practising circular mobilities to access alternative economic opportunities reveal their ‘rootedness’ to land, community, and local culture, which instils an aspiration to return despite livelihood pressures in origin areas (Blondin 2021; Zickgraf 2022). Therefore, despite being mobile actors, migrants may remain ‘moored’ or ‘rooted’ in their origin areas. In the second instance, migration processes could lead to becoming financially, socially, or psychologically ‘trapped’ in destination areas. For example, people practising labour migration overseas to support declining livelihoods at home were found to become ‘trapped’ in exploitative working conditions and lacking the financial resources to return (Ayeb-Karlsson and Uy 2022). This suggests that mobility experiences may be characterised by both movement and stasis, with migrants experiencing involuntary immobility in their destination areas and return migration processes influenced by staying aspirations.

## Discussion

This review shows that a key focus in environmental immobility scholarship has been to uncover causal relationships between environmental change, various socioeconomic vulnerabilities, and immobility decisions. It finds that there is now a proliferation of studies that have addressed this research gap, highlighting that environmental threats do not shape immobility outcomes unilaterally but in concert with pre-existing economic, political, social, and demographic inequalities and vulnerabilities. While this predominant focus on causality has helped explore the multiple factors shaping immobility outcomes across settings, analytical thinking about the various immobility responses remains underdeveloped.^25^

**Fig. 6:**
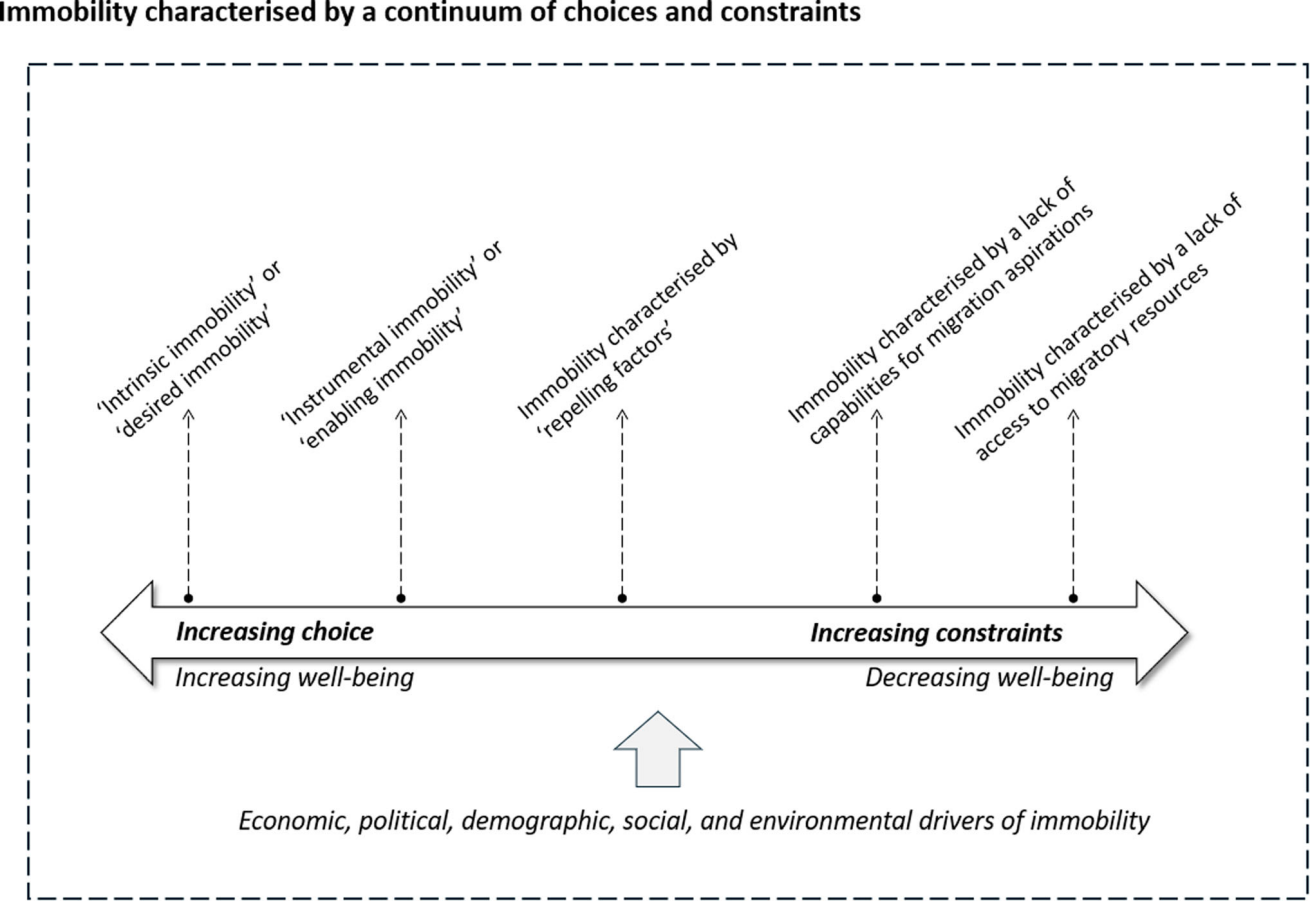
Schematic framework showing immobility outcomes along a continuum of choices and constraints and corresponding implications for stayers’ well-being, using analytical categories from migration theory

The review additionally shows that environmental immobility outcomes are characterised in the empirical literature based on a dichotomous understanding of agency, as either voluntary or involuntary. However, such dichotomisation of voluntariness and involuntariness may be difficult to apply in real-world situations (de Haas 2021; Carling 2024), particularly in contexts of slow-onset environmental change, where people experience a combination of both choices and constraints in their migration or staying decisions.^26^ This means that both ‘voluntary’ and ‘involuntary’ stayers may be faced with a continuum of choices and constraints, acknowledging which allows for a nuanced and more realistic understanding of immobility processes (de Haas 2021; Zickgraf 2022). Instead of binary distinctions between voluntary and involuntary immobility, we may characterise immobility processes along a continuum of intermedial categories, which have corresponding implications for stayers’ well-being^27^ (Fig. 6).

It is possible to categorise the various choices and constraints characterising immobility processes by drawing on a range of analytical categories from migration theory. For instance, the notions of ‘intrinsic’ and ‘instrumental’ value in immobility (or mobility) processes allow us to differentiate between different kinds of immobility choices based on the function they serve in enhancing people’s well-being (de Haas 2021). Immobility holds *instrumental value* when people undertake it for functional reasons (or as the means to other ends), e.g. people staying put to fulfil familial obligations and for supporting the migratory projects of other household members. In these instances, immobility serves certain objectives at the societal and household levels, but their impacts on stayers’ well-being are largely context-dependent. The reviewed literature shows that while immobility undertaken by certain household members (typically female) to enable the mobility of others can help diversify income sources in the face of risks, thereby enhancing overall household resilience and positively contributing to stayers’ well-being (Zickgraf 2022; Khan et al. 2024), it may also lead to poor health outcomes and poverty among stayers in the event of disrupted remittances and reduced livelihoods (Bhatta et al. 2015; Ahmed and Eklund 2021). In contrast, immobility bears *intrinsic value* in instances where people undertake it as an end in itself and to partake in associated experiences, e.g. people staying in place due to place-based ties and benefits. Being intrinsically valuable, these immobility decisions directly enhance stayers’ well-being.

Similar to notions of ‘instrumental’ and ‘intrinsic’ values of immobility, migration scholarship points to different ‘ways’ of staying put (Mata-Codesal 2015) based on unequal relations of power within households and communities (Jónsson 2011; Mata-Codesal 2018). Scholars distinguish between ‘desired immobility’ and ‘enabling immobility’ where the former represents staying practices to benefit from location-specific ‘insider advantages’ such as upward social mobility, while the latter constitutes immobility processes that are undertaken to support relatives’ migratory projects, e.g. women taking care of productive and reproductive tasks in the household while men migrate.^28^ Their work reveals that immobility processes are shaped by stayers’ expected roles in mobility processes specific to the sociocultural contexts under consideration.

Other conceptual readings of immobility distinguish between ‘retaining’ and ‘repelling’ aspects of staying aspirations (Schewel 2015, 2020). Similar to ‘intrinsic’ notions of immobility and ‘desired immobility’, authors suggest ‘retaining’ factors to be location-specific benefits that motivate people to remain in place, such as maintaining kinship ties and religious affiliations; conversely, ‘repelling’ factors diminish migration intentions due to negative perceptions about migration journeys and destinations. ‘Retaining’ factors are analytically distinguishable from ‘repelling’ factors for staying put, with the former adding value to people’s quality of life, while the latter may have diminishing impacts on stayers’ well-being. Empirical literature reviewed here reveals that apprehensions about undertaking migration lead people to remain under life-threatening conditions in hazard-prone settings with diminished access to viable livelihoods (Adams 2016; Mohammadi and Khanian 2023). These apprehensions may result from sociopsychological constraints such as adverse experiences in destination areas (Mohammadi and Khanian 2023; Wyngaarden et al. 2023), negative feedback from migrant networks, or psychological distress (Ayeb-Karlsson et al. 2020).

Migration scholarship also points to a category of stayers who remain in place because they lack the capability to develop migration aspirations (Schewel 2015, 2020). Termed acquiescent immobility (with ‘acquiescent’ implying the acceptance of constraints), this describes the preference to stay among those who have no real ability to migrate due to a lack of migratory resources. Falling in between a continuum of choices and constraints, acquiescent immobility represents a null choice for remaining in place even at the expense of diminished well-being. Other than a lack of economic or social resources to undertake migration, acquiescent immobility may also result from entrenched societal norms inhibiting mobility. For instance, the empirical literature shows how women living in deeply unequal societies may elect to stay put even during life-threatening hazard events due to an internalised religious and moral value system that discourages them from venturing outside their homes (Ayeb-Karlsson 2020a, 2020c).

People staying put due to a lack of migratory resources required for undertaking migration likely face the highest constraints in their immobility and mobility decisions. These constraints may arise from people’s lack of access to ‘economic (material), social (other people), cultural (ideas, knowledge and skills) and bodily (good health, physical condition and habitus) resources’ needed for undertaking migration (de Haas 2021, 15). Political and legal constraints such as restrictive migration policies also diminish people’s ability to migrate (Schewel 2020). In the reviewed literature, studies find people practising climate-sensitive livelihoods (Mallick et al. 2023), those with limited social networks (Thiede and Brown 2013), and poor physical and mental health (Ayeb-Karlsson et al. 2020; Blondin 2020) to have diminished abilities to undertake migration. People ‘trapped’ in environmentally fragile locations due to low migratory resources that constrain their ability to undertake migration are arguably faced with the most adverse consequences for their well-being.

While the aspiration-capability framework groups all stayers with the intention and ability to stay in place as ‘voluntary’, the proposed approach identifies intermedial categories of voluntariness. Unpacking the aspiration to stay allows us to better understand the contextual features of staying decisions. For instance, the three categories of immobility towards the voluntary end of the spectrum (Fig. 6), i.e. those remaining put due to place-based advantages and positive well-being impacts, those who stay to fulfil a strategic role, e.g. undertaking place-based tasks that support ex-situ migratory projects, and those who stay because they are apprehensive about the rewards of migration are qualitatively different in terms of the extent of choice available to stayers and consequences for their well-being. Similarly, factors that constrain movement, such as internalised value systems or low resources that prohibit the *development* of mobility intentions, are qualitatively different from the lack of access to economic or social capital preventing people from realising their relocation *intentions*. By highlighting these interstitial ways of staying put, this approach points to a nuanced understanding of voluntariness and involuntariness in immobility decision-making.

Other than a deeper understanding of agency, environmental immobility processes can also be characterised by their spatial and temporal dimensions (Schewel 2020). Pertinent questions to ask may be to what spatial extent people remain put in environmentally high-risk locations, for instance, whether people continue to remain in affected homes and homesteads, relocate to surrounding lesser affected areas, or relocate farther away from sources of environmental harm but within administrative boundaries of the village or city or geographically bounded units, such as islands. The immobility experiences of people residing in highly exposed housing, for instance, to riverbank erosion, may be different from those having the means to rebuild houses in less exposed inland areas. Accordingly, immobility experiences can also vary temporally among those staying put in affected regions, for instance, over multiple generations, for entire lifetimes, or relatively shorter durations interspersed with circular mobilities. Understanding why certain households stay put in high-risk locations over generations or for entire lifetimes while others in the same region and exposed to similar adverse environmental impacts stay put for shorter periods may be crucial for uncovering various perceptions of environmental threats and differing motives for staying in place.

### Policy implications

Architects of the ‘trapped populations’ concept initially argued that involuntary immobility in the face of risks is likely to result in humanitarian crises needing urgent policy action (Foresight 2011). However, environmental immobility has garnered limited attention within global migration and climate policy regimes compared to mobility outcomes thus far (Naser et al. 2023). For instance, within the UN climate regime, the 2011 Cancun Agreement (UNFCCC 2011) and the subsequently constituted Warsaw International Mechanism (WIM) for Loss and Damage primarily focus on addressing the migration, displacement, and relocation outcomes of at-risk communities (Ayeb‐Karlsson et al. 2022). Similarly, in the Global Compact for Safe, Orderly and Regular Migration (UNGA 2019, 10), UN Member States highlight ‘the potential implications [of environmental and climatic change] for migration, while recognising that adaptation in the country of origin [is] a priority’. This draws only a cursory attention to the adaptation needs of immobile place-based communities. Regional policy frameworks in Latin America and the Pacific address the precarity of stayers through human rights frameworks and disaster risk reduction approaches while laying greater emphasis on mobility outcomes (Thornton et al. 2023). Human rights approaches such as the recently recognised right to a healthy environment (UNGA 2022) accords substantive (resource-based) and procedural (justice-based) rights to a clean, healthy and sustainable environment to all members of the international community; however, it remains to be integrated into domestic regimes that can tangibly support the staying intentions of communities affected by environmental stress.

Given limited policy attention on the topic, researchers (Naser et al. 2023) viewing immobility in terms of the binary categories of voluntary and involuntary propose a two-pronged policy approach: in-situ adaptation to climatic risks for voluntary stayers and planned relocation of ‘trapped’ communities. However, as this review suggests, immobility decision-making may involve both choices and constraints, unpacking the plurality of which can help develop corresponding policy measures. While adapting to climatic risks through livelihood diversification and risk reduction approaches can support all groups of stayers at either end of the spectrum of (in)voluntariness (Fig. 6), additional policy measures by governments and non-governmental organisations are needed to address the vulnerabilities characterising intermedial immobility categories. For instance, government interventions to enhance social justice and equality among marginalised sections and address gender hierarchies can alter intrahousehold power structures that adversely impact those practising ‘enabling immobility’. This can reduce mobility constraints arising from entrenched gender hierarchies in societies that discourage the mobility of women and relegate them to homebound tasks that are potentially detrimental to their freedom and well-being (see Vigil 2024). Secondly, facilitating safe and dignified opportunities for labour migration from rural settings facing climate change-induced livelihood pressures can help those practising immobility due to ‘repelling factors’ (i.e. due to apprehensions surrounding migration processes) realise their migration intentions and support place-based income sources. This involves ensuring safe access to external sources of employment and addressing issues of abusive working and living conditions in urban destinations. Finally, access to relocation and migratory resources can support those lacking the capability to move elsewhere. However, this not only includes the material resources required for such a move, e.g. financial resources and alternative housing, but also extends to creating viable livelihood opportunities in destination areas. As the reviewed literature points out, rural agrarian communities often lack the necessary skills that would equip them to work in non-farming sectors characteristic of urban areas (Rabbani et al. 2022). Furthermore, relocation initiatives need to be grounded in subjectively determined conceptions of risks and habitability^29^ (Gini et al. 2024) and actively involve affected communities to ensure just outcomes (Bertana 2020).

## Conclusion

The ‘trapped populations’ thesis in the Foresight Report (2011) drew attention to the diverse ways in which environmental change impacts human society. It found that aside from triggering the forced migration of people, environmental stress coupled with low economic resources, can inhibit mobility from high-risk settings. This systematic review of empirical research on environmental immobility reveals that there is now a proliferation of studies addressing the multiple societal factors influencing immobility decisions in the face of risks. Studies were found to largely focus on immobility drivers based on a dichotomous characterisation of immobility as either forced or voluntary. This review argues that while we have a rich understanding of immobility drivers, the characterisation of environmental immobility processes lacks analytical nuance. Drawing on theoretical insights from migration studies, it advances a schematic framework that can help us appreciate how staying in place involves a continuum of choices and constraints, resulting in intermedial immobility categories that better represent the plurality and complexity of staying decisions. Future studies can use the framework provided here to further analyse the context-specific features of immobility processes in the face of risks.

## Supplementary Information

Below is the link to the electronic supplementary material.

Supplementary file1 (PDF 240 KB)

## Data Availability

The author confirms that all data generated or analysed during this study are included in this article and the supplementary material.

## Footnotes

1 Environmental migration scholarship shows that people may have a greater ability to exercise choice by using migration as a possible adaptive response in slow-onset events, e.g. by diversifying income sources (Black et al. 2011a), however, it also points to the possible maladaptive nature of such migration practices (see Vinke et al. 2020 for an overview).

2 See the edited issue by Erdal and Oeppen (2018) and Kollar and Boucher (2023) for conceptual discussions on the notion of voluntariness in the broader migration studies field, where scholars argue that migration (or staying) processes may be deemed voluntary only when people have access to subjectively determined and acceptable alternatives. However, these conceptual articulations of voluntariness, and relatedly coercion, have not yet percolated into scholarship on environmental (im)mobility decision-making.

3 This article uses the phrase ‘environmental change’ to denote both natural and human processes triggering changes in the natural environment and avoids the debate of whether human mobility or immobility processes are particularly attributable to climatic change.

4 While scholars resist the separation between the broader migration studies research field and the niche area of environmental migration research and call for greater integration between the two (Zickgraf 2021; de Sherbinin et al. 2022), the distinction between the two fields is made here to highlight the differing trajectories of research on immobility.

5 The differentiation between voluntary and involuntary immobility based on an assessment of people’s aspirations and capabilities for migration was developed as a heuristic device for explaining instances of failed migration, in contrast to prevailing theories suggesting high global mobility (Carling 2002). However, this divide between voluntary and involuntary migration (and immobility) has often been uncritically adopted by subsequent studies, with migration scholars calling for more nuanced understandings of choice and coercion in mobility and immobility processes (see Erdal and Oeppen 2018; de Haas 2021; Ottonelli and Torresi 2023; Carling 2024).

6 Both the terms ‘environmental/climate immobility’ and ‘environmental non-migration’ have been used so far in the environmental migration research field to depict people staying in place despite environmental threats. Here, the term ‘environmental immobility’ is used for two reasons: First, recent scholarship shows that the immobility of people in environmentally high-risk regions is not equivalent to stasis or non-migration, as staying in place may also involve small-scale mobilities that sustain translocal livelihoods (Wiegel et al. 2019; McMichael et al. 2021). Second, immobility scholarship within migration studies similarly points to the fact that people may practice a range of movements while maintaining spatial continuity in one’s place of residence. Besides, immobility is a wider term than non-migration, with the latter defining those who stay ‘by what they are not’ (Schewel 2020; Salazar 2021). Nevertheless, the search criteria used in the literature search strategy accounts for all terminological variations of environmental immobility (see Table 1).

7 Peer-reviewed journal articles were used, as opposed to non-peer-reviewed literature such as book chapters, editorials, policy documents, and conference proceedings, as a proxy for scientific quality.

8 See similar search strategies concerning the base year in discursive reviews on ‘trapped populations’ in Ayeb-Karlsson et al. (2018) and Ayeb‐Karlsson et al. (2022).

9 Publications of the UN Framework Convention for Climate Change (UNFCCC 2012) and the World Meteorological Organisation (WMO 2021) were consulted to ensure that the search terms used to represent environmental and climatic change comprised a comprehensive list of extreme weather events and slow-onset environmental change. To represent human immobility processes, terminologies used in the two research fields of environmental migration and migration studies (Zickgraf 2018; Schewel 2020; Gruber 2021) were used.

10 It is important to note that limiting search results to peer-reviewed journal articles and those in the English language may restrict the generalisability of conclusions reached in the study.

11 Accordingly, four groups of articles were excluded: (i) those which deal with the broader climate change adaptation and migration research fields but do not address immobility, (ii) those which primarily focus on migration outcomes and place only a secondary or subsidiary focus on immobility, (iii) those drawing a comparison between factors influencing decisions to migrate or stay during disaster events without an in-depth consideration of the latter, and (iv) those reporting the same case study findings in more than one publication (in this case, only one was retained to avoid duplication).

12 Accordingly, articles without empirical content, i.e. with solely theoretical or policy-related content, were excluded.

13 Adding relevant literature from reference lists of search results constitutes a supplementary search strategy in systematic reviews (Papaioannou et al. 2010), which can help overcome limitations posed by the possible non-indexation of studies in bibliographic databases (Gusenbauer and Haddaway 2020; Delgado-Quirós et al. 2024). Articles included at this stage were put through the same screening and eligibility assessment processes applied to the inital search results from the two bibliographic databases.

14 The thematic classification of the empirical literature into studies addressing various factors of immobility follows from the multicausality thesis in the broader environmental migration research field which suggests that mobility (and immobility) outcomes of environmental change are mediated by the non-environmental context, i.e. a range of pre-existing economic, political, social, and demographic factors in a given setting (Black et al. 2011b).

15 By ‘non-environmental’ factors, authors refer to those not directly linked with biophysical or climatic risks. See Obokata et al. (2014), where authors similarly highlight the need to understand the ‘non-environmental’ drivers, i.e. historical and political influencing factors, of international environmental migration.

16 While there is a rise in the number of studies between 2020 and 2022 (see Fig. 2), this upward trend in empirical outputs relatively declines in 2023 but remains higher than the pre-2020 years, which saw only up to two studies per year. Since the literature search was conducted in February 2024, the entire year’s output could not be accounted for, thereby making the number of publications in 2024 appear much lower. Whether the number of outputs indeed declines or plateaus following this relative decline in outputs in 2023 is a subject worth considering in future reviews on the topic.

17 To provide context to the large number of studies conducted in Bangladesh, it is important to point out that several studies in the dataset stem from the same research projects, such as the outputs from a project led by Mallick and colleagues that examines voluntary immobility in rural coastal regions in Bangladesh (e.g. Mallick et al. 2020; Biswas and Mallick 2021; Mallick et al. 2022; Mallick et al. 2023) and those from a project led by Ayeb-Karlsson and colleagues that explores involuntary immobility in rural (Ayeb-Karlsson 2020a; Ayeb-Karlsson b) and urban settings in Bangladesh (Ayeb-Karlsson et al. 2020; Ayeb-Karlsson 2020c).

18 See a review paper by Castillo Betancourt and Zickgraf (2024) for a recent exploration of this issue, where the differences in societal norms regarding gender relations lead to very different mobility and immobility outcomes across regional settings.

19 Examples from coastal and island settings include those in Bangladesh (e.g. Ahmed and Eklund 2021; Rabbani et al. 2022), Senegal (Zickgraf 2019; Zickgraf 2022), Indonesia (Amin et al. 2021), Fiji (e.g. Piggott-McKellar and McMichael 2021; Yee et al. 2022a), Tuvalu (McMichael et al. 2021), Guinea-Bissau (Santos and Mourato 2024), the Philippines (Ayeb-Karlsson and Uy 2022), Australia (Haynes et al. 2018), and the USA (e.g. Klopfer 2017; Otsuyama et al. 2021).

20 Examples from riparian settings include those in Bangladesh (Paul et al. 2020), India (Bhatta et al. 2015; Sengupta and Samanta 2022), and Zimbabwe (Mavhura 2023).

21 Examples from inland settings include those in Morocco (Van Praag 2021; Van Praag 2022; Ou-Salah et al. 2024), Honduras (Wyngaarden et al. 2023), Iran (Mohammadi and Khanian 2023), and Nigeria (Makanju and Uriri 2021).

22 Examples from mountain settings include those in India (Rai 2022; Upadhyay et al. 2024), Nepal (Bhusal et al. 2021), Pakistan (Cook and Butz 2015; Khan et al. 2024), Tajikistan (Blondin 2020; Blondin 2021; Blondin 2022), Chile (Wiegel et al. 2021), Peru (Adams 2016), Guatemala (Milan and Ruano 2014).

23 Involuntary immobility has been variously characterised in the reviewed literature as ‘trapped populations’ (Adams 2016; Nawrotzki and DeWaard 2018; Ayeb-Karlsson 2020c), ‘resource-constrained immobility’ (Benveniste et al. 2022), and ‘strandedness’ (Cook and Butz 2015).

24 Additionally, some studies do not characterise immobility processes as either involuntary or voluntary (marked as ‘uncategorised’ in Table 3). These consider the immobility experiences of those who lack migration aspirations due to their limited capabilities to migrate, such as women engaged in poorly remunerative subsistence agriculture with gendered household responsibilities (Van Praag 2022; Ou-Salah et al. 2024). Immobility outcomes among people who lack the ability and, therefore, the aspiration to migrate are conceptualised as acquiescent immobility in migration studies (Schewel 2015; Schewel 2020). However, this conceptual category has not duly percolated into environmental immobility research and represents a gap between the two research fields.

25 For instance, Black and colleagues (2011b, S5) suggest that their framework which outlines the multiple environmental and societal drivers of migration does not provide theoretical explanations of migration outcomes, but instead ‘encompass[es] the range of drivers that might affect the volume, direction and frequency of migratory movements, as well as the different levels of analysis at which migration might be considered’. Therefore, their framework helps us better understand the *influencing factors* of (im)mobility but does not provide theoretical explanations for the *differential nature of immobility responses.*

26 While this does not readily apply to contexts of sudden-onset events where the ‘need’ or urgency to flee life-threatening hazards precludes the option to remain, thereby rendering moot any question of exercising agency (Black and Collyer 2014), research concludes that in cases of slow-onset environmental changes, people have greater scope for exercising their choice in migration and staying decisions (Black et al. 2011a).

27 Here, a broad connotation of the notion of well-being is implied which goes beyond the indicators of physical and mental health to encompass people’s ability to pursue their subjective notions of a ‘good life’ rooted in emic understandings of a ‘life worth living’ (Mata-Codesal 2019). For a conceptual overview of how mobility and immobility processes relate to this wider conception of well-being as people’s freedoms to fulfil (broader) life aspirations, see de Haas (2021).

28 By supporting and facilitating the migratory projects of household members, ‘enabling’ stayers may also serve a strategic role in strengthening household resilience to environmental pressures on place-based livelihoods.

29 Subjective conceptions of risks may be related to how staying (and the potential need for relocation) is viewed across settings, based on emic conceptions of a ‘good life’ (Mata-Codesal 2019). Therefore, exploring subjective narratives on immobility and mobility in the context of the broader life aspirations of people, traditional power structures in society, and historical experiences of communities allows for more grounded understandings of immobility decisions (see also Ayeb-Karlsson 2020b). This calls for using qualitative approaches involving long-term engagement with fieldwork, such as through ethnographic and participatory methods (and potentially, with the assistance of a cultural broker), to garner situated understandings of people’s relations with place, community, and local value systems, and relatedly, their staying intentions.

## References

[CR1] Adams, H. 2016. Why populations persist: Mobility, place attachment and climate change. *Population and Environment* 37: 429–448. 10.1007/s11111-015-0246-3.

[CR2] Ahmed, S. and E. Eklund. 2021. Climate change impacts in coastal Bangladesh: Migration, gender and environmental justice. *Asian Affairs* 52: 155–174. 10.1080/03068374.2021.1880213.

[CR3] Ahsan, M.N., F. Khatun, P. Kumar, R. Dasgupta, B.A. Johnson, and R. Shaw. 2022. Promise, premise, and reality: The case of voluntary environmental non-migration despite climate risks in coastal Bangladesh. *Regional Environmental Change*. 10.1007/s10113-021-01864-1.

[CR4] Alam, A. and M.B. Khalil. 2022. Gender, (im)mobility and social relations shaping vulnerabilities in coastal Bangladesh. *International Journal of Disaster Risk Reduction* 82: 103342. 10.1016/j.ijdrr.2022.103342.

[CR5] Amin, C., S. Sukamdi, and R. Rijanta. 2021. Exploring migration hold factors in climate change hazard-prone area using grounded theory study: Evidence from Coastal Semarang Indonesia. *Sustainability (Switzerland)* 13: 4335. 10.3390/su13084335.

[CR6] Ayeb-Karlsson, S. 2020a. I do not like her going to the shelter: Stories on gendered disaster (im)mobility and wellbeing loss in coastal Bangladesh. *International Journal of Disaster Risk Reduction* 50: 101904. 10.1016/j.ijdrr.2020.101904.

[CR7] Ayeb-Karlsson, S. 2020b. No power without knowledge: A discursive subjectivities approach to investigate climate-induced (Im)mobility and wellbeing. *Social Sciences* 9: 103. 10.3390/SOCSCI9060103.

[CR8] Ayeb-Karlsson, S. 2020c. When the disaster strikes: Gendered (im)mobility in Bangladesh. *Climate Risk Management* 29: 100237. 10.1016/j.crm.2020.100237.

[CR9] Ayeb-Karlsson, S. 2020d. When we were children we had dreams, then we came to Dhaka to survive: Urban stories connecting loss of wellbeing, displacement and (im)mobility. *Climate and Development* 13: 348–359. 10.1080/17565529.2020.1777078.

[CR10] Ayeb-Karlsson, S. and N. Uy. 2022. Island stories: Mapping the (im)mobility trends of slow onset environmental processes in three island groups of the Philippines. *Humanities and Social Sciences Communications*. 10.1057/s41599-022-01068-w.

[CR11] Ayeb-Karlsson, S., C.D. Smith, and D. Kniveton. 2018. A discursive review of the textual use of ‘trapped’ in environmental migration studies: The conceptual birth and troubled teenage years of trapped populations. *Ambio* 47: 557–573. 10.1007/s13280-017-1007-6. 29435732 10.1007/s13280-017-1007-6 PMC6072639

[CR12] Ayeb-Karlsson, S., D. Kniveton, and T. Cannon. 2020. Trapped in the prison of the mind: Notions of climate-induced (im)mobility decision-making and wellbeing from an urban informal settlement in Bangladesh. *Palgrave Communications* 6: 15. 10.1057/s41599-020-0443-2.

[CR13] Ayeb-Karlsson, S., A.W. Baldwin, and D. Kniveton. 2022. Who is the climate-induced trapped figure? *WIREs Climate Change* 13: e803. 10.1002/wcc.803.

[CR14] Baer, R.D., S.C. Weller, and C. Roberts. 2019. The role of regional cultural values in decisions about Hurricane evacuation. *Human Organization* 78: 133–146. 10.17730/0018-7259.78.2.133.

[CR15] Balgah, R.A., and J.N. Kimengsi. 2022. A review of drivers of environmental non-migration decisions in Africa. *Regional Environmental Change* 22: 125. 10.1007/S10113-022-01970-8.

[CR16] Bell, A.R., D.J. Wrathall, V. Mueller, J. Chen, M. Oppenheimer, M. Hauer, H. Adams, S. Kulp, et al. 2021. Migration towards Bangladesh coastlines projected to increase with sea-level rise through 2100. *Environmental Research Letters* 16: 024045. 10.1088/1748-9326/ABDC5B. 36034333 10.1088/1748-9326/abdc5b PMC9415774

[CR17] Benveniste, H., M. Oppenheimer, and M. Fleurbaey. 2022. Climate change increases resource-constrained international immobility. *Nature Climate Change* 12: 634–641. 10.1038/s41558-022-01401-w.

[CR18] Berrang-Ford, L., J.D. Ford, and J. Paterson. 2011. Are we adapting to climate change? *Global Environmental Change* 21: 25–33. 10.1016/j.gloenvcha.2010.09.012. 10.1016/j.gloenvcha.2010.05.003 PMC7125589 32288342

[CR19] Bertana, A. 2020. The role of power in community participation: Relocation as climate change adaptation in Fiji. *Environment and Planning C: Politics and Space* 38: 902–919. 10.1177/2399654420909394.

[CR20] Bhatta, G.D., P.K. Aggarwal, S. Poudel, and D.A. Belgrave. 2015. Climate-induced migration in South Asia: Migration decisions and the gender dimensions of adverse climatic events. *The Journal of Rural and Community Development* 10: 1–23.

[CR21] Bhusal, P., J.N. Kimengsi, and K. Raj Awasthi. 2021. What drives environmental (Non-)migration around the Himalayan Region? Evidence from rural Nepal. *World Development Perspectives* 23: 100350. 10.1016/j.wdp.2021.100350.

[CR22] Biswas, B. and B. Mallick. 2021. Livelihood diversification as key to long-term non-migration: Evidence from coastal Bangladesh. *Environment, Development and Sustainability* 23: 8924–8948. 10.1007/s10668-020-01005-4.

[CR23] Black, R., S.R.G. Bennett, S.M. Thomas, and J.R. Beddington. 2011a. Migration as adaptation. *Nature* 478: 447–449. 10.1038/478477a. 22012304 10.1038/478477a

[CR24] Black, R., W.N. Adger, N.W. Arnell, S. Dercon, A. Geddes, and D. Thomas. 2011b. The effect of environmental change on human migration. *Global Environmental Change* 21: S3–S11. 10.1016/J.GLOENVCHA.2011.10.001.

[CR25] Black, R. and M. Collyer. 2014. Populations “trapped” at times of crisis. *Forced Migration Review* 45. Retrieved 16 March 2025 from https://www.fmreview.org/black-collyer/.

[CR26] Blondin, S. 2020. Understanding involuntary immobility in the Bartang Valley of Tajikistan through the prism of motility. *Mobilities* 15: 543–558. 10.1080/17450101.2020.1746146.

[CR27] Blondin, S. 2021. Staying despite disaster risks: Place attachment, voluntary immobility and adaptation in Tajikistan’s Pamir Mountains. *Geoforum* 126: 290–301. 10.1016/j.geoforum.2021.08.009.

[CR28] Blondin, S. 2022. Let’s hit the road! Environmental hazards, materialities, and mobility justice: Insights from Tajikistan’s Pamirs. *Journal of Ethnic and Migration Studies* 48: 3416–3432. 10.1080/1369183X.2022.2066261.

[CR29] Boas, I., H. Wiegel, C. Farbotko, J. Warner, and M. Sheller. 2022. Climate mobilities: Migration, im/mobilities and mobility regimes in a changing climate. *Journal of Ethnic and Migration Studies* 48: 3365–3379. 10.1080/1369183X.2022.2066264.

[CR30] Boas, I., N. de Pater, and B.T. Furlong. 2023. Moving beyond stereotypes: The role of gender in the environmental change and human mobility nexus. *Climate and Development* 15: 1–9. 10.1080/17565529.2022.2032565.

[CR31] Braun, V. and V. Clarke. 2021. *Thematic analysis: A practical guide*. London: SAGE Publications.

[CR32] Carling, J. 2002. Migration in the age of involuntary immobility: Theoretical reflections and cape Verdean experiences. *Journal of Ethnic and Migration Studies* 28: 5–42. 10.1080/13691830120103912.

[CR33] Carling, J. 2024. Why do people migrate? Fresh takes on the foundational question of migration studies. *International Migration Review* 58: 1757–1791. 10.1177/01979183241269445. 10.1177/01979183241269445 PMC13032834 41909486

[CR34] Carling, J. and K. Schewel. 2018. Revisiting aspiration and ability in international migration. *Journal of Ethnic and Migration Studies* 44: 945–963. 10.1080/1369183X.2017.1384146.

[CR36] Castillo Betancourt, T. and C. Zickgraf. 2024. It’s not just about women: broadening perspectives in gendered environmental mobilities research. *Climate and Development*: 1–22. 10.1080/17565529.2024.2393305.

[CR37] Chumky, T., M. Basu, K. Onitsuka, M.L. Raihan, and S. Hoshino. 2023. How do left-behind families adapt to the salinity-induced male out-migration context? A case study of Shyamnagar Sub-District in coastal Bangladesh. *Sustainability (Switzerland)* 15: 2756. 10.3390/su15032756.

[CR38] Cook, N. and D. Butz. 2015. The dialectical constitution of mobility and immobility: Recovering from the Attabad Landslide disaster, Gojal, Gilgit-Baltistan, Pakistan. *Contemporary South Asia* 23: 388–408. 10.1080/09584935.2015.1090950.

[CR39] Cundill, G., C. Singh, W.N. Adger, R. Safra de Campos, K. Vincent, M. Tebboth, and A. Maharjan. 2021. Toward a climate mobilities research agenda: Intersectionality, immobility, and policy responses. *Global Environmental Change* 69: 102315. 10.1016/j.gloenvcha.2021.102315.

[CR200] Czaika, M., and C. Reinprecht. 2022. Why do people stay put in environmentally stressful regions? Cognitive bias and heuristics in migration decision-making. *Regional Environmental Change* 22. 10.1007/s10113-022-01934-y

[CR201] Debray, A., I. Ruyssen, and K. Schewel. 2023. The aspiration to stay: A global analysis. *International Migration Review*. 10.1177/01979183231216087

[CR41] de Haas, H. 2021. A theory of migration: The aspirations-capabilities framework. *Comparative Migration Studies*. 10.1186/s40878-020-00210-4. 10.1186/s40878-020-00210-4 PMC7902564 33680858

[CR42] de Sherbinin, A., K. Grace, S. McDermid, K. van der Geest, M.J. Puma, and A. Bell. 2022. Migration theory in climate mobility research. *Frontiers in Climate*. 10.3389/fclim.2022.882343.

[CR43] UNFCCC. 2011. Report of the Conference of the Parties on its sixteenth session, held in Cancun from 29 November to 10 December. CP.2010/7/Add. 1. Retrieved 16 March 2025 from https://unfccc.int/resource/docs/2010/cop16/eng/07a01.pdf.

[CR44] Delgado-Quirós, L., I.F. Aguillo, A. Martín-Martín, E.D. López-Cózar, E. Orduña-Malea, and J.L. Ortega. 2024. Why are these publications missing? Uncovering the reasons behind the exclusion of documents in free-access scholarly databases. *Journal of the Association for Information Science and Technology* 75: 43–58. 10.1002/asi.24839.

[CR45] Erdal, M.B. and C. Oeppen. 2018. Forced to leave? The discursive and analytical significance of describing migration as forced and voluntary. *Journal of Ethnic and Migration Studies* 44: 981–998. 10.1080/1369183X.2017.1384149.

[CR46] Farbotko, C. and C. McMichael. 2019. Voluntary immobility and existential security in a changing climate in the Pacific. *Asia Pacific Viewpoint* 60: 148–162. 10.1111/apv.12231.

[CR47] Ferris, E. and S. Weerasinghe. 2020. Promoting human security: planned relocation as a protection tool in a time of climate change. *Journal on Migration and Human Security* 8: 134–149. 10.1177/2331502420909305.

[CR48] Foresight. 2011. *Migration and global environmental change: Future challenges and opportunities. Final Project Report.* London. Retrieved 16 March 2025 from https://assets.publishing.service.gov.uk/media/5a74b18840f0b61df4777b6c/11-1116-migration-and-global-environmental-change.pdf.

[CR49] Ghosh, R.C. and C. Orchiston. 2022. A systematic review of climate migration research: Gaps in existing literature. *SN Social Sciences* 2: 47. 10.1007/s43545-022-00341-8.

[CR50] Gini, G., A. Piggott-Mckellar, H. Wiegel, F. Neu, A.-C. Link, C. Fry, T. Tabe, O. Adegun, et al. 2024. Navigating tensions in climate change-related planned relocation. *Ambio* 53: 1262–1266. 10.1007/s13280-024-02035-2. 38847970 10.1007/s13280-024-02035-2 PMC11300726

[CR51] Gruber, E. 2021. Staying and immobility: New concepts in population geography? A literature review. *Geographica Helvetica* 76: 275–284. 10.5194/gh-76-275-2021.

[CR52] Gusenbauer, M. and N.R. Haddaway. 2020. Which academic search systems are suitable for systematic reviews or meta-analyses? Evaluating retrieval qualities of Google Scholar, PubMed, and 26 other resources. *Research Synthesis Methods* 11: 181–217. 10.1002/jrsm.1378. 31614060 10.1002/jrsm.1378 PMC7079055

[CR53] Haynes, K., M. Tofa, A. Avci, J. van Leeuwen, and L. Coates. 2018. Motivations and experiences of sheltering in place during floods: Implications for policy and practice. *International Journal of Disaster Risk Reduction* 31: 781–788. 10.1016/j.ijdrr.2018.07.011.

[CR54] Jónsson, G. 2011. *Non-migrant, sedentary, immobile, or “left behind”? Reflections on the absence of migration*. IMI Working Papers Series 2011, No. 39. Oxford.

[CR55] Khan, S.A., M. Doevenspeck, and O. Sass. 2024. Climate (im)mobilities in the Eastern Hindu Kush: The case of Lotkuh Valley Pakistan. *Population and Environment* 46: 2. 10.1007/s11111-023-00443-2.

[CR56] Khatun, F., M.N. Ahsan, S. Afrin, J. Warner, R. Ahsan, B. Mallick, and P. Kumar. 2022. Environmental non-migration as adaptation in hazard-prone areas: Evidence from coastal Bangladesh. *Global Environmental Change* 77: 102610. 10.1016/j.gloenvcha.2022.102610.

[CR57] Klopfer, A.N. 2017. Choosing to stay: Hurricane Katrina narratives and the history of claiming place-knowledge in New Orleans. *Journal of Urban History* 43: 115–139. 10.1177/0096144215576332.

[CR58] Kollar, E. and F. Boucher. 2023. Introduction: Voluntariness and migration. *Ethics and International Affairs* 37: 401–405. 10.1017/S0892679423000400.

[CR59] Makanju, A.O.M. and A.E. Uriri. 2021. Aging, resilience, and migration in the Sudano-Sahelian ecological belt in Nigeria. *AHMR African Human Mobility Review* 7: 7–25.

[CR60] Mallick, B. 2023. Environmental non-migration: Analysis of drivers, factors, and their significance. *World Development Perspectives* 29: 100475. 10.1016/j.wdp.2022.100475.

[CR61] Mallick, B. and L. Hunter. 2023. Environmental non-migration: Framework, methods, and cases. *Regional Environmental Change* 23: 22. 10.1007/S10113-022-02019-6. 38343651 10.1007/s10113-022-02019-6 PMC10854421

[CR62] Mallick, B. and L.M. Hunter. 2024. Environmental migration and non-migration: Learning through an intergenerational lens. *Migration Studies* 12: mnae031. 10.1093/migration/mnae031.

[CR63] Mallick, B. and J. van den Berg. 2025. Intergenerational grounding of women’s environmental non-migration. *Population and Environment* 47: 7. 10.1007/s11111-025-00475-w. 40123945 10.1007/s11111-025-00475-w PMC11928378

[CR64] Mallick, B., Z. Sultana, and C.M. Bennett. 2020. How do sustainable livelihoods influence environmental (non-)migration aspirations? *Applied Geography* 124: 4718. 10.1016/j.apgeog.2020.102328.

[CR65] Mallick, B., K.G. Rogers, and Z. Sultana. 2022. In harm’s way: Non-migration decisions of people at risk of slow-onset coastal hazards in Bangladesh. *Ambio* 51: 114–134. 10.1007/s13280-021-01552-8. 33825159 10.1007/s13280-021-01552-8 PMC8651874

[CR66] Mallick, B., C. Priovashini, and J. Schanze. 2023. I can migrate, but why should I?—voluntary non-migration despite creeping environmental risks. *Humanities and Social Sciences Communications*. 10.1057/s41599-023-01516-1.

[CR67] Mallick, B. and J. Schanze. 2020. Trapped or voluntary? Non-migration despite climate risks. *Sustainability (Switzerland)* 12: 4718. 10.3390/SU12114718.

[CR68] Mata-Codesal, D. 2015. Ways of staying put in Ecuador: Social and embodied experiences of mobility-immobility interactions. *Journal of Ethnic and Migration Studies* 41: 2274–2290. 10.1080/1369183X.2015.1053850.

[CR69] Mata-Codesal, D. 2018. Is it simpler to leave or to stay put? Desired immobility in a Mexican village. *Population, Space and Place*. 10.1002/PSP.2127.

[CR70] Mata-Codesal, D. 2019. Aspiring to a life worth living: Some considerations about the aspirations to migrate or stay put. In *renewing the migration debate. Building disciplinary and geographical bridges to explain global migration*, ed. S. Vezzoli and H. de Haas, 46–52. Amsterdam: KNAW Academy Colloquium.

[CR71] Mavhura, E. 2023. Why do riparian communities persist in disaster-prone areas? Empirical evidence from Mbire District, Zimbabwe. *Environment, Development and Sustainability* 27: 4831–4847. 10.1007/s10668-023-04102-2.

[CR72] McAdam, J., ed. 2010. *Climate change and displacement: multidisciplinary perspectives*. Oxford and Portland, Oregon: Hart Publishing.

[CR73] McLeman, R. and F. Gemenne, eds. 2018. *Routledge handbook of environmental displacement and migration*. Abdingon: Routledge.

[CR74] McMichael, C., C. Farbotko, A. Piggott-McKellar, T. Powell, and M. Kitara. 2021. Rising seas, immobilities, and translocality in small island states: Case studies from Fiji and Tuvalu. *Population and Environment* 43: 82–107. 10.1007/s11111-021-00378-6.

[CR75] Milan, A. and S. Ruano. 2014. Rainfall variability, food insecurity and migration in Cabricán, Guatemala. *Climate and Development* 6: 61–68. 10.1080/17565529.2013.857589.

[CR76] Mohammadi, S. and M. Khanian. 2023. Staying in crisis: Choice or coercion a review of the reasons of rural-to-urban migrations due to environmental changes in Iranian villages. *Space and Culture* 26: 598–617. 10.1177/12063312211018396.

[CR77] Moher, D., A. Liberati, J. Tetzlaff, D.G. Altman, and P. Group. 2009. Preferred Reporting Items for Systematic Reviews and Meta-Analyses: The PRISMA Statement. *Annals of Internal Medicine* 151: 264–269. 10.1371/journal.pmed.1000097 10.7326/0003-4819-151-4-200908180-00135 19622511

[CR78] Mortreux, C., S. Jarillo, J. Barnett, and E. Waters. 2023. Climate change and migration from atolls? No evidence yet. *Current Opinion in Environmental Sustainability* 60: 101234. 10.1016/j.cosust.2022.101234.

[CR79] Nabong, E.C., L. Hocking, A. Opdyke, and J.P. Walters. 2023. Decision-making factor interactions influencing climate migration: A systems-based systematic review. *WIREs Climate Change* 14: e828. 10.1002/wcc.828.

[CR80] Nahin, K.T.K., S.B. Islam, S. Ahmed, M.S. Mondal, S.B. Murshed, and S. Nowreen. 2023. Voluntary immobility despite hazard: A case of Jamuna floodplain in Bangladesh. *GeoJournal* 88: 3497–3514. 10.1007/s10708-022-10820-3.

[CR81] Naser, M.M., B. Mallick, R. Priodarshini, S. Huq, and A. Bailey. 2023. Policy challenges and responses to environmental non-migration. *npj Climate Action*. 10.1038/s44168-023-00033-w.

[CR82] Nawrotzki, R.J. and J. DeWaard. 2018. Putting trapped populations into place: Climate change and inter-district migration flows in Zambia. *Regional Environmental Change* 18: 533–546. 10.1007/s10113-017-1224-3. 29456454 10.1007/s10113-017-1224-3 PMC5810408

[CR83] Obokata, R., L. Veronis, and R. McLeman. 2014. Empirical research on international environmental migration: A systematic review. *Population and Environment* 36: 111–135. 10.1007/s11111-014-0210-7. 25132701 10.1007/s11111-014-0210-7 PMC4131126

[CR84] Otsuyama, K., E. Dunn, C. Bell, and N. Maki. 2021. Typology of human mobility and immobility for disaster risk reduction: Exploratory case study in Hillsborough county Florida. *Natural Hazards Review* 22: 05021010. 10.1061/(ASCE)NH.1527-6996.0000498.

[CR85] Ottonelli, V. and T. Torresi. 2023. Voluntariness and migration: A restatement. *Ethics and International Affairs* 37: 406–426. 10.1017/S0892679423000424.

[CR86] Ou-Salah, L., L. Van Praag, and G. Verschraegen. 2024. Household gender roles and slow-onset environmental change in Morocco: A barrier or driver to develop migration aspirations? *Journal of Development Studies* 60: 309–323. 10.1080/00220388.2023.2279486.

[CR87] Papaioannou, D., A. Sutton, C. Carroll, A. Booth, and R. Wong. 2010. Literature searching for social science systematic reviews: Consideration of a range of search techniques. *Health Information and Libraries Journal* 27: 114–122. 10.1111/j.1471-1842.2009.00863.x. 20565552 10.1111/j.1471-1842.2009.00863.x

[CR88] Paul, B.K., M.K. Rahman, T. Crawford, S. Curtis, M.G. Miah, M.R. Islam, and M.S. Islam. 2020. Explaining mobility using the community capital framework and place attachment concepts: A case study of riverbank erosion in the lower Meghna Estuary Bangladesh. *Applied Geography* 125: 102199. 10.1016/j.apgeog.2020.102199.

[CR89] Pemberton, G., T. Furlong, S. Pemberton, B. Tripathy Furlong, O. Scanlan, V. Koubi, M. Guhathakurta, M. Khalid Hossain, et al. 2021. Staying as climate change adaptation strategy: A proposed research agenda. *Geoforum* 121: 192–196. 10.1016/j.geoforum.2021.02.004.

[CR90] Piggott-McKellar, A.E. and C. McMichael. 2021. The immobility-relocation continuum: Diverse responses to coastal change in a small island state. *Environmental Science and Policy* 125: 105–115. 10.1016/j.envsci.2021.08.019.

[CR91] Piguet, E., R. Kaenzig, and J. Guélat. 2018. The uneven geography of research on environmental migration. *Population and Environment* 39: 357–383. 10.1007/S11111-018-0296-4.

[CR92] Rabbani, M.M.G., M. Cotton, and R. Friend. 2022. Climate change and non-migration—exploring the role of place relations in rural and coastal Bangladesh. *Population and Environment* 44: 99–122. 10.1007/s11111-022-00402-3. 35615058 10.1007/s11111-022-00402-3 PMC9123852

[CR93] Rai, A. 2022. Chasing the ghosts: Stories of people left behind on the frontline of climate and ecological crisis. *South African Journal of Psychology* 52: 460–471. 10.1177/00812463221130902.

[CR94] Salazar, N.B. 2021. Immobility: The relational and experiential qualities of an ambiguous concept. *Transfers* 11: 3–21. 10.3167/TRANS.2021.110302.

[CR95] Santos, C. and J.M. Mourato. 2024. I was born here, I will die here: Climate change and migration decisions from coastal and insular Guinea-Bissau. *Geografiska Annaler, Series B: Human Geography* 106: 137–155. 10.1080/04353684.2022.2154689.

[CR96] Schewel, K. 2020. Understanding immobility: Moving beyond the mobility bias in migration studies. *International Migration Review* 54: 328–355. 10.1177/0197918319831952.

[CR97] Schewel, K. 2015. Understanding the aspiration to stay: A case study of young adults in Senegal. International Migration Institute (IMI) Working Paper Series.

[CR98] Schreier, M. 2014. Qualitative content analysis. In *The SAGE handbook of qualitative data analysis*, ed. U. Flick, 170–183. London: SAGE Publications Ltd.

[CR99] Sengupta, A. and G. Samanta. 2022. Understanding immobility of a highly vulnerable coastal village in the Indian Sundarban. *Regional Environmental Change* 22: 90. 10.1007/s10113-022-01931-1.

[CR100] Sheller, M. and J. Urry. 2006. The new mobilities paradigm. *Environment and Planning A* 38: 207–226. 10.1068/A37268.

[CR101] Stark, O. and D.E. Bloom. 1985. The new economics of labor migration. *American Economic Review* 75: 173–178.

[CR202] Stockdale, A. and T. Haartsen. 2018. Editorial introduction: Putting rural stayers in the spotlight. *Population, Space and Place* 24: e2124. 10.1002/psp.2124

[CR102] Straehle, C. 2023. Migration, climate change, and voluntariness. *Ethics and International Affairs* 37: 452–469. 10.1017/S0892679423000412.

[CR103] Tanner, T., D. Lewis, D. Wrathall, R. Bronen, N. Cradock-Henry, S. Huq, C. Lawless, R. Nawrotzki, et al. 2014. Livelihood resilience in the face of climate change. *Nature Climate Change* 5: 23–26. 10.1038/nclimate2431.

[CR104] Thiede, B.C. and D.L. Brown. 2013. Hurricane Katrina: Who stayed and why? *Population Research and Policy Review* 32: 803–824. 10.1007/s11113-013-9302-9.

[CR105] Thornton, F., D. Andreolla Serraglio, and A. Thornton. 2023. Trapped or staying put: Governing immobility in the context of climate change. *Frontiers in Climate*. 10.3389/fclim.2023.1092264.

[CR106] Tripathy Furlong, B., H. Adams, I. Boas, J. Warner, and H. Van Dijk. 2022. Gendered (im)mobility: Emotional decisions of staying in the context of climate risks in Bangladesh. *Regional Environmental Change* 22: 123. 10.1007/s10113-022-01974-4.

[CR107] UNFCCC. 2012. Slow-Onset Events: Technical Paper FCCC/TP/2012/7. Retrieved 16 March 2025 from https://unfccc.int/documents/7429.

[CR108] UNGA. 2019. Global compact for safe, orderly and regular migration. A/RES/73/195. Retrieved 16 March 2025 from https://digitallibrary.un.org/record/1660537?ln=en&v=pdf.

[CR109] UNGA. 2022. The human right to a clean, healthy and sustainable environment. A/RES/76/300. Retrieved 16 March 2025 from https://digitallibrary.un.org/record/3983329?ln=en&v=p.

[CR110] Upadhyay, H., K. Vinke, and H. Weisz. 2024. We are still here climate change, gender and immobility in highly mobile Himalayan communities. *Climate and Development* 16: 443–457. 10.1080/17565529.2023.2230176.

[CR111] Van Praag, L. 2021. Can I move or can I stay? Applying a life course perspective on immobility when facing gradual environmental changes in Morocco. *Climate Risk Management* 31: 100274. 10.1016/j.crm.2021.100274.

[CR112] Van Praag, L. 2022. Gender, environmental change, and migration aspirations and abilities in tangier and tinghir, Morocco. *Human Ecology* 50: 23–34. 10.1007/s10745-021-00296-z.

[CR113] Vigil, S. 2024. Towards a feminist political ecology of migration in a changing climate. *Geoforum* 155: 104076. 10.1016/j.geoforum.2024.104076.

[CR114] Vinke, K., J. Bergmann, J. Blocher, H. Upadhyay, and R. Hoffmann. 2020. Migration as adaptation? *Migration Studies* 8: 626–634. 10.1093/migration/mnaa029.

[CR115] Warner, K. and T. Afifi. 2014. Where the rain falls: Evidence from 8 countries on how vulnerable households use migration to manage the risk of rainfall variability and food insecurity. *Climate and Development* 6: 1–17. 10.1080/17565529.2013.835707.

[CR116] Wiegel, H., I. Boas, and J. Warner. 2019. A mobilities perspective on migration in the context of environmental change. *WIREs Climate Change* 10: e610. 10.1002/wcc.610.

[CR117] Wiegel, H., J. Warner, I. Boas, and M. Lamers. 2021. Safe from what? Understanding environmental non-migration in Chilean Patagonia through ontological security and risk perceptions. *Regional Environmental Change* 21: 43. 10.1007/s10113-021-01765-3.

[CR118] WMO. 2021. WMO Atlas of mortality and economic losses from weather, climate and water extremes (1970–2019). Geneva.

[CR119] Wyngaarden, S., S. Humphries, K. Skinner, E. Lobo Tosta, V. Zelaya Portillo, P. Orellana, and W. Dodd. 2023. You can settle here: Immobility aspirations and capabilities among youth from rural Honduras. *Journal of Ethnic and Migration Studies* 49: 212–231. 10.1080/1369183X.2022.2031922.

[CR120] Yee, M., A.E. Piggott-McKellar, C. McMichael, and K.E. McNamara. 2022. Climate change, voluntary immobility, and place-belongingness: Insights from Togoru Fiji. *Climate* 10: 46. 10.3390/cli10030046.

[CR121] Yee, M., K.E. McNamara, A.E. Piggott-McKellar, and C. McMichael. 2022. The role of Vanua in climate-related voluntary immobility in Fiji. *Frontiers in Climate*. 10.3389/fclim.2022.1034765.

[CR122] Zickgraf, C. 2018. Immobility. In *Routledge handbook of environmental displacement and migration*, ed. R. McLeman and F. Gemenne, 71–84. London: Routledge.

[CR123] Zickgraf, C. 2019. Keeping people in place: Political factors of (im)mobility and climate change. *Social Sciences* 8: 228. 10.3390/socsci8080228.

[CR124] Zickgraf, C. 2021. Theorizing (im)mobility in the face of environmental change. *Regional Environmental Change*. 10.1007/S10113-021-01839-2.

[CR125] Zickgraf, C. 2022. Relational (im)mobilities: A case study of Senegalese coastal fishing populations. *Journal of Ethnic and Migration Studies* 48: 3450–3467. 10.1080/1369183X.2022.2066263.

