# Supplementary material for: Environmental immobility: A systematic review of empirical research
Source: Ambio. 2025 Jun 11;54(11):1729–56. doi: 10.1007/s13280-025-02195-9 (PMC12480326; doi:10.1007/s13280-025-02195-9)
Supplement: Supplementary file 1 — Supplementary file1 (PDF 240 KB) [file 13280_2025_2195_MOESM1_ESM.pdf]

Ambio

Supplementary Information

This supplementary information has not been peer reviewed.

Title: "Environmental immobility: a systematic review of empirical research"

Limits applied during the search process

The search strategy involved using the Boolean search terms (see Table 1 and section 'Review methodology' in the main text) with limits applied to the source and document type, language, and subject areas of articles (Table S1).

Table S1. Limits applied during the search process.

Limits applied	Included	Excluded
**Source type**	Journal	Books, book series, conference proceedings
**Document type**	Article	Review article, letter, note, editorial, conference paper, shot survey, conference review, erratum, proceeding papers
**Language**	English	All other languages
**Subject areas**	Environmental and social sciences* (e.g., 'environmental science', 'earth and planetary sciences', 'social sciences', 'arts and humanities', 'psychology', 'economics, econometrics and finance', 'decision sciences'; 'environmental sciences ecology', 'water resources', 'geography', 'physical geography', 'social sciences', 'public administration', 'demography', 'development studies', 'psychology', 'government law', 'oceanography', 'urban studies', 'area studies', 'anthropology', 'behavioural sciences', 'remote sensing', 'sociology' 'arts and humanities', 'ethnic studies', 'cultural studies', 'mathematical methods in social sciences', 'social issues', 'international relations', 'family studies', 'women's studies', 'social work')	Unrelated subject areas from the natural, engineering, and medical sciences

* The subject areas were deliberately kept broad to ensure a broad coverage, e.g., 'physical geography' and 'earth and planetary sciences' are slightly distant topics, but these were still included to ensure that all relevant articles would be found. The subsequent screening process eliminated unrelated articles as described in the section 'Review methodology'.
